# Measuring engagement among older adults using a multidimensional approach to communication

**DOI:** 10.3389/fpsyg.2022.981008

**Published:** 2022-11-21

**Authors:** Madeleine Jessica Radnan, Weicong Li, Catherine J. Stevens, Clair Hill, Caroline Jones

**Affiliations:** The MARCS Institute for Brain, Behaviour and Development, Western Sydney University, Penrith, NSW, Australia

**Keywords:** engagement, communication, method, older adult, behaviour, affect, verbal, non-verbal

## Abstract

Characterizing older adult engagement is important to determine the effectiveness of interventions. Engagement refers to the occupying of oneself in external stimuli and is observable across multiple dimensions of behavior. Engagement of older adults is commonly investigated using a single behavioral dimension. There is a dearth of analytical methods that can simultaneously quantify both verbal and non-verbal forms of communication as proxies for engagement. In this article, we present a multidimensional technique to measure engagement of older adults using techniques appropriate for people with varying degrees of dementia. The new analytical approach measures facial movement, lexical use, and prosodic patterns of speech as indices of affective and behavioral outcomes of engagement. Contexts for engagement included a dyadic reminiscence therapy interview and a 12-week technology-driven group reminiscence therapy. Illustrative examples of the technique are described by two participants from two different groups in a naturalistic setting. Application of these analytical techniques can enhance measurement precision and further develop the science and evidence base, especially for, but not confined to, non-pharmacological interventions.

## Introduction

Understanding engagement is important for assessing the impact and effectiveness of interventions. Engagement has been defined as ‘the act of being occupied or involved with an external stimulus, which includes concrete objects, activities, and other persons’ (Cohen-Mansfield et al., 2010). Behavior as an indicator of engagement is motivated by internal factors such as cognition and emotion (Cohen-Mansfield et al., 2009; Skinner et al., 2009; Cohen-Mansfield et al., 2011).

Behavior and affect have been proposed as measurable outcomes of group engagement in the Comprehensive Process Model of Group Engagement (CPMGE) framework (Cohen-Mansfield et al., 2017). Within the CPMGE, the complex relationship and interaction between person, stimuli, and environmental attributes influence the behavior (talking, agitation, restlessness, smiling, movement, etc.) and affect (positive, negative) of older adults. Being able to detect and measure the affective and behavioral outcomes of engagement can provide an understanding of the impact of recreational, social and care activities in residential care, among other applications.

Both spoken word (lexical) use and prosodic patterns of speech can be considered when measuring verbal communication as an outcome of engagement. Lexical use is related to the words used within the dialogue. Analysis of lexical use (e.g., personal pronouns) can indicate the personalized nature of speech and a marker for the focus on self and others (Davis and Brock, 1975; Small et al., 1998). That is, if a person uses more words such as ‘I’, ‘we’, and ‘our’, it can indicate that what is being said may relate to a personal story or experience, thereby sharing identity and personal history. Measuring the affective semantics of words in speech and their relationship to one another can also give insight into the affective state of the person (Borelli et al., 2018). For example, the word ‘good’ is classified as a positive emotive word and can indicate the participant’s positive affect concerning the event they are discussing.

Prosodic features of speech reflect the acoustic characteristics of speech and include utterance timing, intonation, tone, stress, and rhythm. Even though natural variations in speech production occur as people age (Smith et al., 1987), there is a greater impairment of prosodic expression in people with dementia. For example, people with Alzheimer’s disease have been shown to have ~30% reduction in the length of utterance produced compared to healthy controls, reflecting a smaller syntactic sentence when speaking (Stickle and Wanner, 2017). Prosodic features can also give insight into the emotional connotations of dialogue (Misiewicz et al., 2018), particularly through pitch contour by measuring the fundamental frequency (Busso et al., 2009). It has been shown, for example, that happy emotions are associated with a higher pitch level and more variability compared to sad emotions, and angry emotions are associated with a faster rate of speech compared to sad emotions (Stolarski, 2015; Juslin et al., 2017). One of the most noteworthy prosodic patterns to distinguish between people with dementia and healthy controls is the standard deviation (SD) of the fundamental frequency (F0), which is a measure of pitch variation. In a study by Gonzalez-Moreira et al. (2015) people with mild dementia (M = 42.0, SD = 13.5) had a greater F0 variability compared to healthy controls (M = 29.3, SD =8.7). In summary, previous research points to the potential utility of lexical and prosodic patterns as measures of engagement for older adults and a new method for doing so will be proposed.

Non-verbal body language is a vital communicative element and is thought to comprise 70% of all communication (Mehraby, 2005). Facial expressions, as a form of non-verbal communication, are one of the most telling markers of affect-driven behavioral engagement (Ekman, 1965). Facial expressions are one of the behavioral means through which emotions are expressed (Ekman, 1965) and are indicative of the behavioral intentions of a person (Fridlund, 1994; Horstmann, 2003). Facial expressions are constructed from combinations of different facial muscle movements. For example, smiling with the lip corner puller muscles combined with the raising of the cheek muscles conveys the facial expression of happiness. Facial movements can be used to quantify facial expressions, to help characterize non-verbal engagement of older adults.

The present analytical approach addresses the challenge of measuring engagement of older adults: our specific setting is psychosocial activities in residential care. We propose an analytical method derived from the psychological and linguistic understanding of communication that quantifies complex affective and behavioral outcomes of engagement. These outcomes of engagement may be used to assess the impact of a range of psychosocial interventions and meaningful activities (Tierney and Beattie, 2020) on people in residential care. The proposed multidimensional approach is important as it will be able to determine the impact of interventions on older adults where there are idiosyncratic characteristics of engagement. This is important as the rate of change in individuals is due more to person-specific factors than a developmental process (Wilson et al., 2002). In brief, this approach addresses both verbal and non-verbal affective and behavioral outcomes of engagement by measuring facial movement, lexical use, and prosodic patterns of speech, as measures of engagement. As this approach is innovative for older adult intervention research, the primary focus of this article is on the method and, in particular, the analytical process. The paper’s emphasis on analysis, analytics, and multidimensional methods is to suit dynamic (changing) and multifaceted conditions within an individual, across time, and across individuals. This is important with such individualization in people being expressed in cognition, memory and function (De Brigard et al., 2022) and with natural differences that arise from culturally and linguistically diverse people (Patel et al., 2022). The proposed multidimensional approach is important as will be able to determine the impact of interventions on older adults where there are idiosyncratic characteristics of engagement. This is important as the rate of change in individuals is due more to person-specific factors than a developmental process (Wilson et al., 2002).The method is innovative in that it involves a multidimensional approach to measuring and characterizing verbal and non-verbal outcomes of engagement that can be used across varying interventions and contexts. This approach contrasts with more common tools that measure engagement, such as *via* researcher observations, surveys, interviews or explicitly active engagement (Ke et al., 2020; Williams et al., 2021; Coley et al., 2022). The latter sometimes appear to lack power and/or suitability for use with older adults who may have limited ability to self-report *via* complex scales or interviews, and/or a person may not be consciously aware of how engaged they are with a situation, event, or experience. As a subtle and precise measure of engagement, the reported analytical method in this paper complements more traditional approaches that examine engagement, such as those through self-reported measures (Goldberg et al., 2002), or the Observational Measurement of Engagement (OME; Cohen-Mansfield et al., 2009).

In this article, we demonstrate the application of the multidimensional approach within an existing reminiscence therapy (RT) intervention study, which is called Time Travelling with Technology (TTT). First, a brief overview of TTT is described. Second, each dimension of engagement is described, and the dependent variables are defined. Third, data preparation from audio-visual recordings is described. Last, two in-depth participant examples from the TTT intervention study are presented to demonstrate the utility of this method.

**Figure 1:**
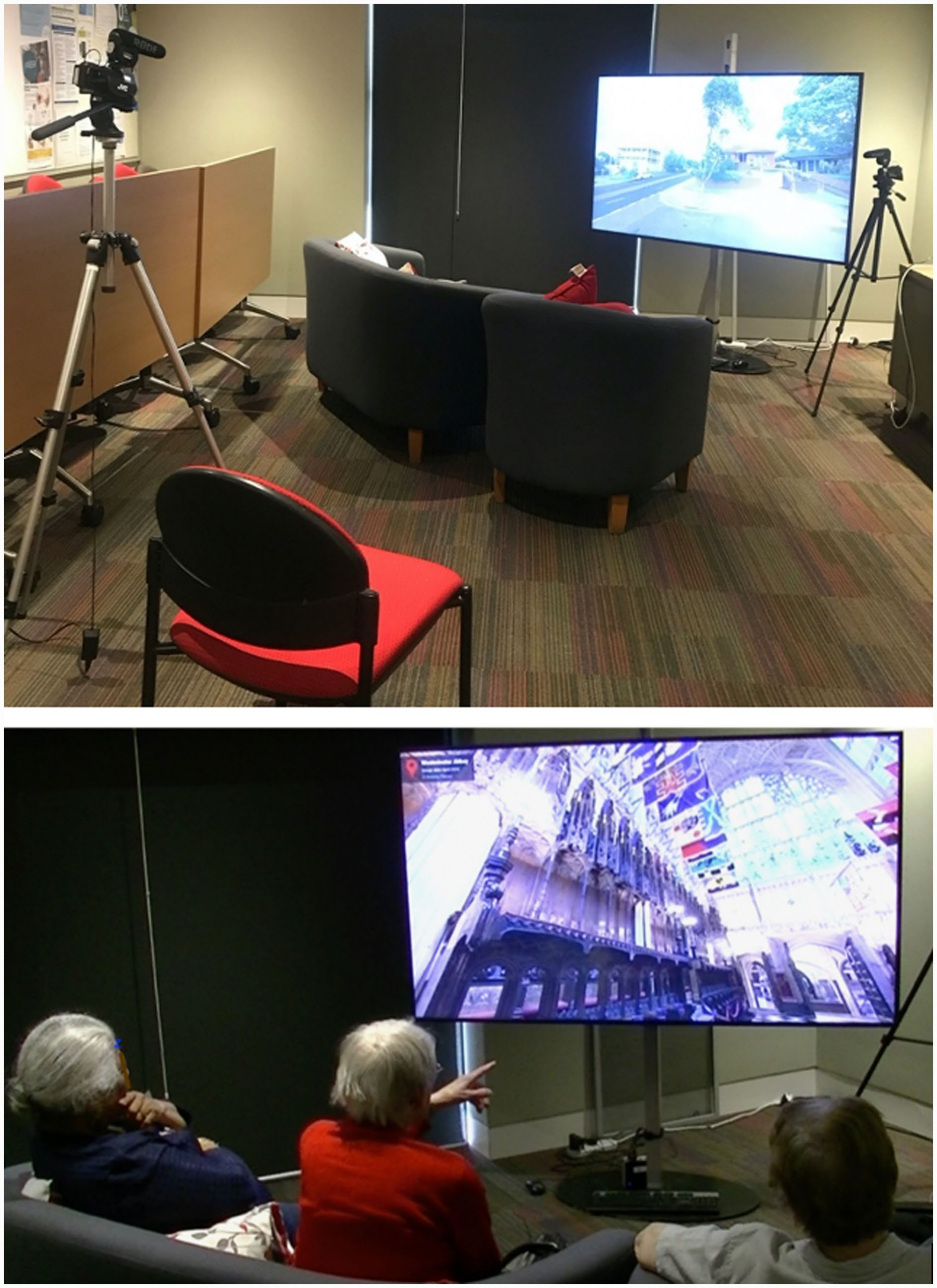
The Time Travelling with Technology (TTT) program environment. The older adults sit in an arc around the television and view locations that are specific to adults in the session. One camera was located next to the screen facing the participants face on, to capture facial movement and audio. A second camera was facing the screen and was situated behind the participants to capture the locations viewed across the session. The facilitator sat on the floor next to the television facing the participants and used a tablet device to drive the TTT display.

In the TTT program, a group of older adults view on a large screen, images of locations they have chosen from their past, sourced from Google Street View and Google Maps. Figure 1 shows the TTT set-up. Participants had diverse cultural backgrounds including Polish, English, Scottish, Sri Lankan, Australian, Croatian, and Samoan. Using a repeated measures design, a Low-tech (LT) and High-tech (HT) conditions each ran for 6 weeks, with a 3-week mid-program break. In the Low-Tech condition (LT), TTT was operationalized as static images of locations. In the High-Tech condition (HT), TTT was operationalized with dynamic and immersive features of the locations. This included the ability to pan around the environment, have a 360-degree view of locations, move up and down streets, and explore the inside of buildings. The conditions were counterbalanced across groups with 5 participants beginning in the LT condition, and 4 participants beginning with the HT condition. The weekly sessions consisted of groups of 2–4 participants for approximately 30 min and involved a form of reminiscence therapy. The procedure also included a dyadic RT interview between the facilitator and older adult at three-time points; pre-, mid-, and post-program. An interview before the study served as a baseline for behavior and affect. During the interview, participants were asked questions about their personal stories across their life.

## Analytical method

**Figure 2:**
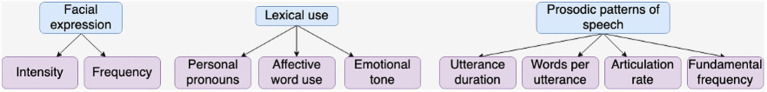
Relationship of affect/behavior measures to their dependent variables.

To measure engagement in older adults, the analytical method draws on three dimensions: facial movement, lexical use, and prosodic patterns of speech. Figure 2 relates each affect/behavior measure to its dependent variables.

### Facial expression as a measure of engagement

Visual recordings of participants during the sessions were used to analyze facial expressions. The Facial Action Coding System (FACS; Ekman and Friesen, 1978) is an anatomically based system that measures and taxonomizes different expressive facial movements. Facial expressions are broken down into combinations of Action Units (AUs) whereby a singular AU may represent the movement of an individual muscle or a group of muscles.

Facial expression AUs were assessed using the OpenFace 2.0 Facial Analysis Toolkit (OpenFace; Baltrušaitis et al., 2018). OpenFace is a Python and Torch-based face recognition software that uses deep neural networks. It has the capability of facial landmark detection, head pose estimation, AU recognition, and eye-gaze estimation. Commonly used facial recognition software is designed to assess a singular face detected by a camera, that captures the full face of the person, has minimal head position movement, and takes up most of the recording screen. This is not ideal when trying to capture the facial expressions of people in a group setting. The benefit of using OpenFace is that it has the capability of detecting faces that are small on the screen and uses real-time pose estimation to track an individual face across frames. Therefore, this software allowed a single camera to record all the participants in the session, face on.

OpenFace recognizes a subset of 18 AUs. The following five AUs listed in Table 1 were used as measures of engagement as they correspond with an array of positive and negative facial expressions.

**Table 1:** The five Action Units (AUs) that were used for analysis.

AU	FACS name	Muscle	Emotions	Example
4	Brow lowerer	Depressor glabellae, depressor supercilii, corrugator supercilii	Sadness, fear, anger, confusion	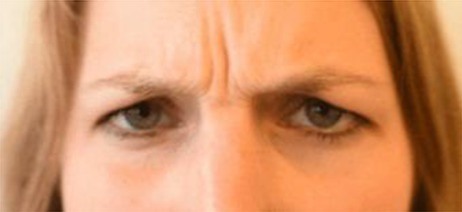
6	Cheek raiser	Orbicularis oculi (pars orbitalis)	Happiness	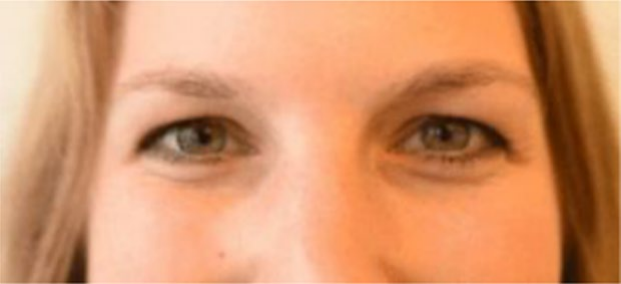
12	Lip corner puller	Zygomaticus major	Happiness, contempt	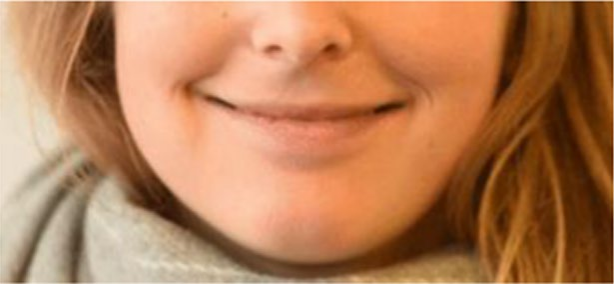
15	Lip corner depressor	Depressor anguli oris (triangularis)	Sadness, disgust, confusion	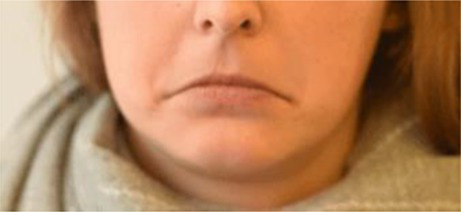
17	Chin raiser	Mentalis	Interest, confusion	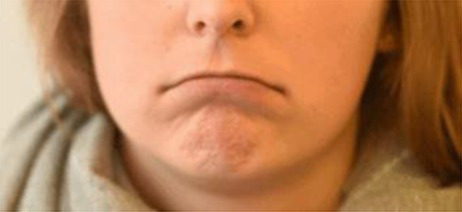

Each AU was chosen as it was associated with a generally clear direction of emotional affect. For each AU the Facial Action Coding System (FACS) name is listed, as well as the muscle involved in the movement and the emotion/s that the AU contributes to. Examples of each action unit are presented. Adapted with permission from imotions by Farnsworth (2018). Available at: https://imotions.com/blog/facial-action-coding-system/.

The OpenFace AU models are trained and evaluated with various databases that are publicly available online (Baltrušaitis et al., 2018). The two properties that describe the AUs are presence and intensity. The presence of an AU is characterized as either 0 (absent) or 1 (present). The intensity of the AU is on a continuous five-point scale from 0 (minimal intensity) to 5 (maximal intensity). Each of the five AUs was analyzed for the percentage of time the AU was activated (presence) during a session and the intensity of the AU when activated.

### Lexical use as a measure of engagement

The Linguistic Inquiry and Word Count (LIWC; Pennebaker et al., 2015) computationally analyzes dialogue into five broad word domains (linguistic dimensions, psychological processes, relativity, personal concerns, and spoken categories), which further divides into 68 subcategories. The LIWC was used to investigate the percentage of total speech that was occupied by personal pronouns and affective words, and to determine the emotional tone of language.

Various lexical markers, see Table 2, were used to index a participant’s sense of self and interlocutors/others (Small et al., 1998; Borelli et al., 2018) and valence of affective speech within a session. As a marker of focus on self and others in the interaction, the first person singular and plural pronouns ‘I’ and ‘we’ were investigated. As a marker of focus on others within the interaction, the second-and third-person pronouns ‘you’, ‘he/she’, and ‘they’ were investigated. The pronouns were pooled together to measure the expression of identities and focus on self and others in interaction. Emotional valence of words, as the valence of affective speech, included positive emotion words and negative emotion words. For example, the words ‘good’, ‘happy’, and ‘pretty’ are positive, and the words ‘hate’, ‘worthless’ and ‘enemy’ are negative. The emotional tone of speech was measured as an emotional valence of speech, on a scale of 0 (most negative) to 100 (most positive). A number lower than 50 is related to a more negative tone displaying greater anxiety, sadness and/or hostility. A number higher than 50 is related to a more positive, upbeat and/or vivid tone. A neutral number around 50 suggests a lack of emotional valence in tone or varying levels of ambivalence, that is, contradictory or mixed feelings (Pennebaker et al., 2015).

**Table 2:** The three lexical markers used for analysis.

LIWC marker	Description	Measure
ppron	Personal pronouns	Percentage of total speech (%)
affect	Words associated with positive and negative emotion	Percentage of total speech (%)
tone	Emotional tone of language	Continuous scale 0 (most negative) – 100 (most positive)

For each marker, the LIWC parameter name is listed, the description of the parameter and the units of measurement.

### Prosodic patterns of speech as a measure of engagement

Prosodic patterns of speech that were measured included mean duration of utterance (seconds), words per utterance, articulation rate (words/s), and pitch as measured through the fundamental frequency (F0), see Table 3. The articulation rate was calculated, to characterize the fluidity of speech as a behavioral marker of engagement. Articulation rate is also a measure of energy expenditure when speaking. F0 variability was investigated as an index of the affect-driven behavioral engagement of a participant. The standard deviation of F0 (Hertz) was used to characterize variability in ‘pitch’.

**Table 3:** The four prosodic patterns used for analysis.

Prosodic marker	Description	Measure
mean_dur_utt	Mean duration of utterance	Seconds (s)
mean_no_wd_per_utt	Mean number of words per utterance	Numeric count
sp_rate	Articulation rate	Words per second (words/s)
F0_sd	Standard deviation of fundamental frequency	Hertz (Hz)

For each measure, the analysis coding name is listed, the description of the measure and the units of measurement.

### Data preparation procedure

Weekly TTT sessions over a 12-week period and 3 dyadic RT interviews were audio-visually recorded. The audio-visual recordings were imported into Adobe Premiere and converted the files to a 1920 × 1,080 MPEG-2 movie file (.mpg) and a 48 kHz, 16-bit waveform audio file (.wav). The.mpg movie file was used for subsequent facial movement analysis.

**Figure 3:**
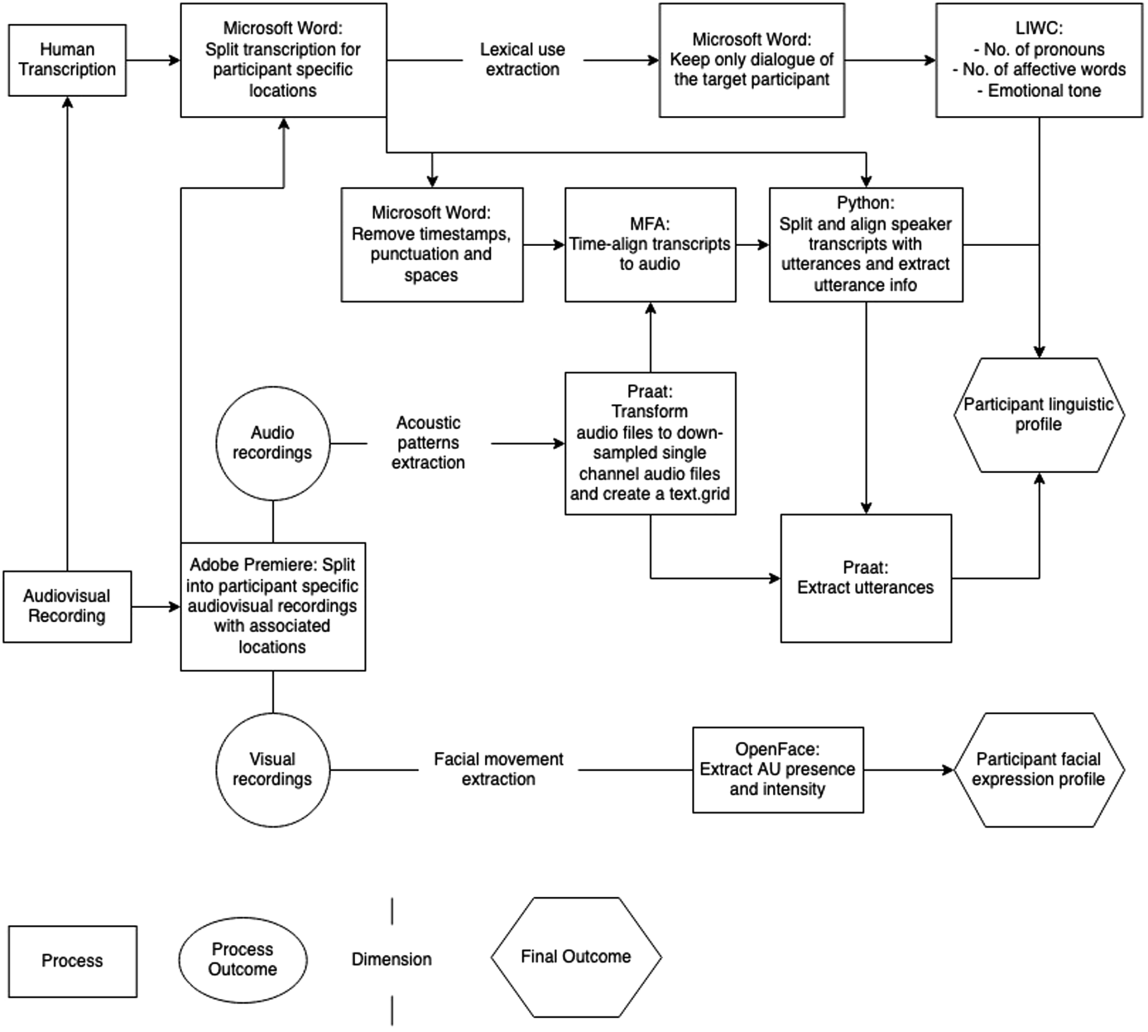
Multidimensional analysis workflow from audio-visual recordings. LIWC = Linguistic Inquiry and Word Count; MFA = Montreal Forced Aligner; AU = action unit.

The.wav audio recordings of sessions were transcribed and saved as Microsoft Word files (.docx), then used for analysis of lexical use and prosodic patterns of speech. Figure 3 represents the analysis workflow.

### Facial movement analysis method

**Figure 4:**
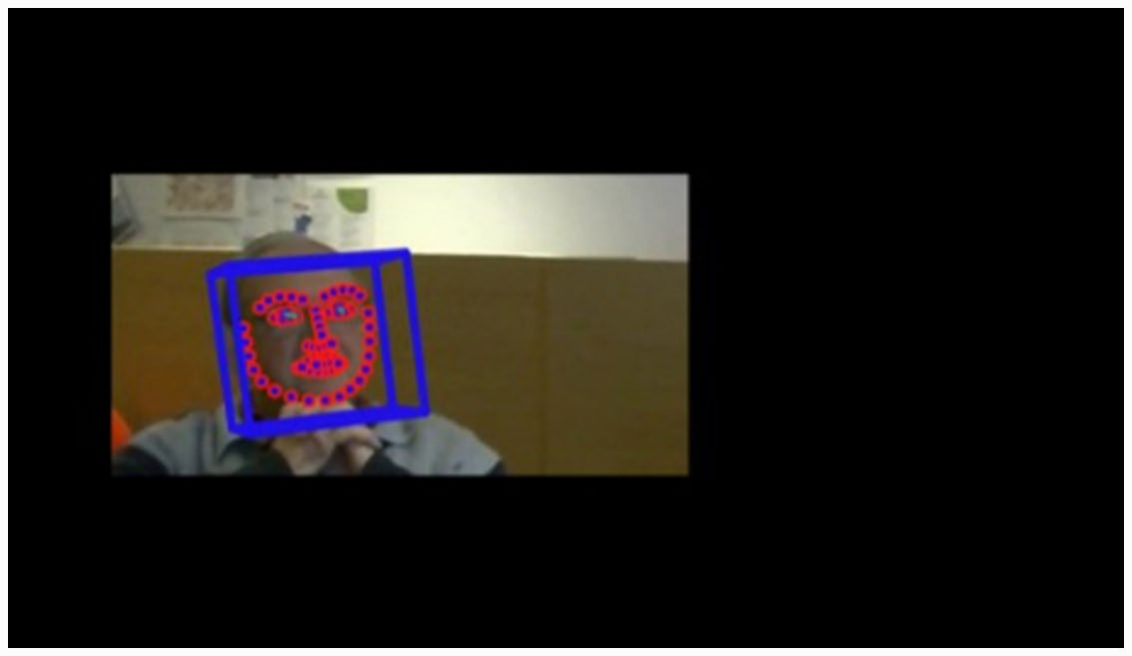
A screenshot of a cropped video recording of a Time Travelling with Technology session being processed through OpenFace.

OpenFace software is a facial recognition program (Baltrušaitis et al., 2018) used to analyze the five dependent variables AUs: 04 – brow lowerer, 06 – cheek raiser, 12 – lip corner puller, 15 – lip corner depressor, and 17 – chin raiser. OpenFace can register the facial properties for multiple people in a frame. It has limitations, however, and is unable to link the individual people to themselves across multiple frames in a video. To be able to analyze the features of an individual participant in a video, individual faces in the video had to be isolated, which was done by cropping the frame and exporting the video with a single cropped face. The output of OpenFace is a comma-separated values file (.csv) that contains rows representing values relating to various facial features at a sampling rate of 0.04 s from the video recording. The processed frames for each participant were manually linked longitudinally over the individual frames within a session and transferred to a master.csv analysis file. A snapshot of the cropped file being processed by OpenFace is shown in Figure 4. Within the image, the red outlined dots represent Facial Landmark Detection, the blue cube represents head pose tracking and the green line represents eye gaze tracking.

The essential columns of the.csv file for the analysis include the ‘frame’, ‘timestamp’, ‘confidence’, ‘success’ and the facial AU columns. ‘Confidence’ is on a scale of 0 to 1 and represents how confident the tracker is in determining the current landmark detection estimate. Any row with a Confidence of <0.8 was deleted. ‘Success’ of the trial and of tracking the facial features is represented as a 1 and an unsuccessful frame is represented as a 0 (for example the head is turned away or is too small). Any row with a success of 0 was deleted.

Each AU presence and intensity column of interest had an averaged value that was calculated. For averaging the intensity, any cell with a ‘0’ under the intensity columns was removed as we were only interested in the intensity response when the movement of the AU was present.

### Lexical use analysis method

The transcripts from the dyadic RT interview and the TTT transcripts included all speakers in the session. The LIWC program analyzes all the text in a file. LIWC cannot identify different speakers in a document. Therefore, to prepare for LIWC processing, it was important to delete all the dialogue that was not the speech of the target participant. The files were then processed in batches by LIWC according to their baseline and relevance of the location viewed to each participant.

The.csv output from LIWC contains columns for the parameters and rows for each file processed. Of the 93 different parameters, the LIWC provides we kept the summary language variables of ‘Tone’ (emotional tone), personal pronouns ‘ppron’ (e.g., ‘I’, ‘we’, ‘you’, ‘she’, ‘he’, ‘they’), and affective processes ‘affect’, which includes both positive and negative emotion words. For ‘ppron’ (personal pronouns) and ‘affect’ (affective processes), the value within each column is the percentage of text that is represented by the parameter. For example, within the column ‘ppron’, a value of 6.47 indicated that 6.47 percent of all the words spoken by the participant were personal pronoun words.

### Prosodic patterns of speech analysis method

In the analysis of prosodic patterns, aligning the transcripts, specific to the participant and their locations during each session, to its audio was the first step and was carried out using the Montreal Forced Aligner (MFA) on a down-sampled (16 kHz) single-channel.wav file with an associated Praat.TextGrid file containing the transcript. The output of the MFA was a new.TextGrid file containing a tier with alignment at the word level. A Python script (based on Python version 2.7; van Rossum et al., 2015 see Supplementary material) 26 reconstructed utterances using aligned words and assign utterances to individual speakers by comparing them with the corresponding transcript. Another Python script (see Supplementary material) extracted measures of prosodic patterns from each speaker.Textgrid to a spreadsheet. The variables measured for each speaker and each session were number of utterances, total duration of utterance, mean duration of utterance, the total number of words, and the mean number of words per utterance. The articulation rate was calculated and manually included in the spreadsheet. Praat extracted the standard deviation of the fundamental frequency (F0), for each speaker’s.Textgrid. To manage typical doubling/halving errors in pitch estimation, the Praat script set the fundamental frequency range for detection to 120–400 Hz (female) and 60–200 Hz (male). The output spreadsheet included duration of utterance in seconds, the mean of the fundamental frequency, the standard deviation of the fundamental frequency, fundamental frequency minimum, and maximum value.

## Illustrations of the behavioral analysis

In this section, we illustrate examples drawing on data from two participants, Angela and Colette (pseudonyms). The descriptive examples show the different engagement dimensions in two contexts [dyadic RT interview and technology-driven group RT (TTT)], across the three behavioral dimensions (facial movement, lexical use, and prosodic patterns).

**Figure 5:**
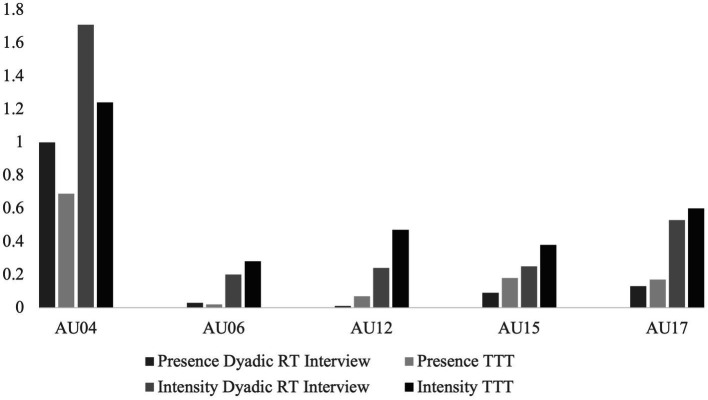
Descriptive statistics of the behavior measures for Angela. Mean of action unit (AU) presence and intensity for Angela across two contexts: dyadic reminiscence therapy (RT) interview, and technology-driven group reminiscence therapy (TTT). The orange and green columns represent the RT context, and the yellow and brown columns represent the TTT context. The presence of the AUs represents the percentage of time the AU was present from 0 (not present) to 1 (always present). The intensity of the AUs represents the intensity of the AU movement on a continuous scale from 0 (min) to 5 (max).

**Figure 6:**
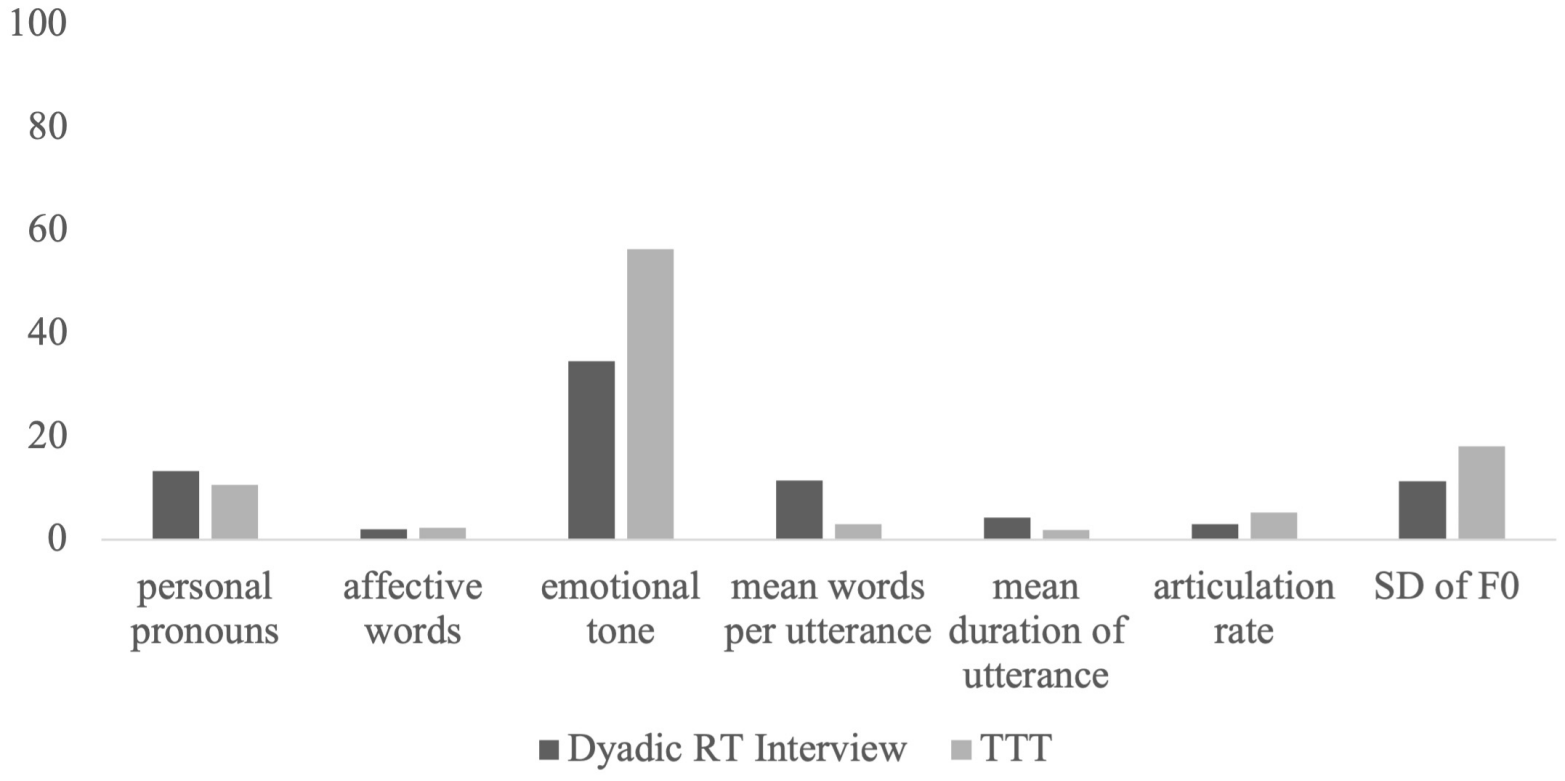
Mean of linguistic outcomes for Angela across two contexts: dyadic reminiscence therapy (RT) interview, and technology-driven group reminiscence therapy (TTT). The use of personal pronouns is measured as a percentage of all speech. Affective words incorporated both positive and negative emotive words and are measured as a percentage of all speech. The emotional tone is measured from 0 (most negative) – 100 (most positive). The mean words per utterance is a numerical count value. The mean duration of utterance is measured in seconds. The articulation rate is the number of words spoken per second calculated from utterances. The standard deviation of fundamental frequency (SD of F0) is measured in Hertz.

Descriptively and as seen in Figure 5, the presence and intensity of AU04 for Angela were greater in the dyadic RT interview compared to the TTT setting. For AU06 there was greater presence of AU06 in the RT interview, however there was a greater intensity of AU06 in the TTT context. For all other AUs, there was greater presence and intensity in the TTT context compared to the dyadic RT interview. Regarding the lexical use outcomes, as seen in Figure 6, Angela had greater personal pronoun use in the RT interview, and greater affective words used with a more positive emotional tone in the TTT context. Regarding the prosodic patterns, there was a greater mean of words per utterance and mean duration of utterance in the dyadic RT interview and a greater articulation rate and F0 variability in the TTT context.

**Figure 7:**
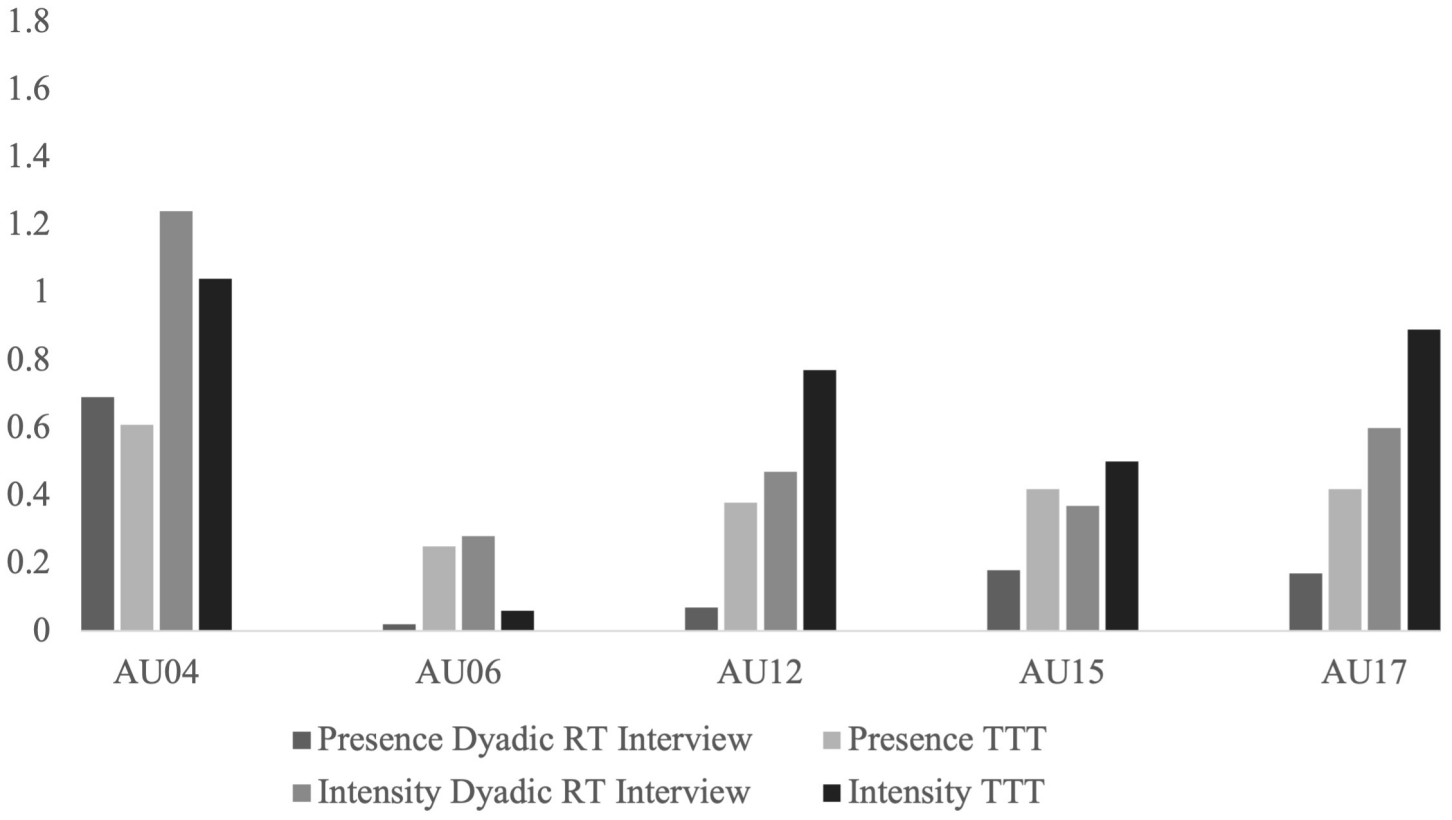
Descriptive statistics of the behavior measures for Colette. Mean of action unit (AU) presence and intensity for Colette across two contexts; dyadic reminiscence therapy (RT) interview, and technology-driven group reminiscence therapy (TTT). The orange and green columns represent the RT context, and the yellow and brown columns represent the TTT context. The presence of the AUs represents the percentage of time the AU was present from 0 (not present) to 1 (always present). The intensity of the AUs represents the intensity of the AU movement on a continuous scale from 0 (min) to 5 (max).

**Figure 8:**
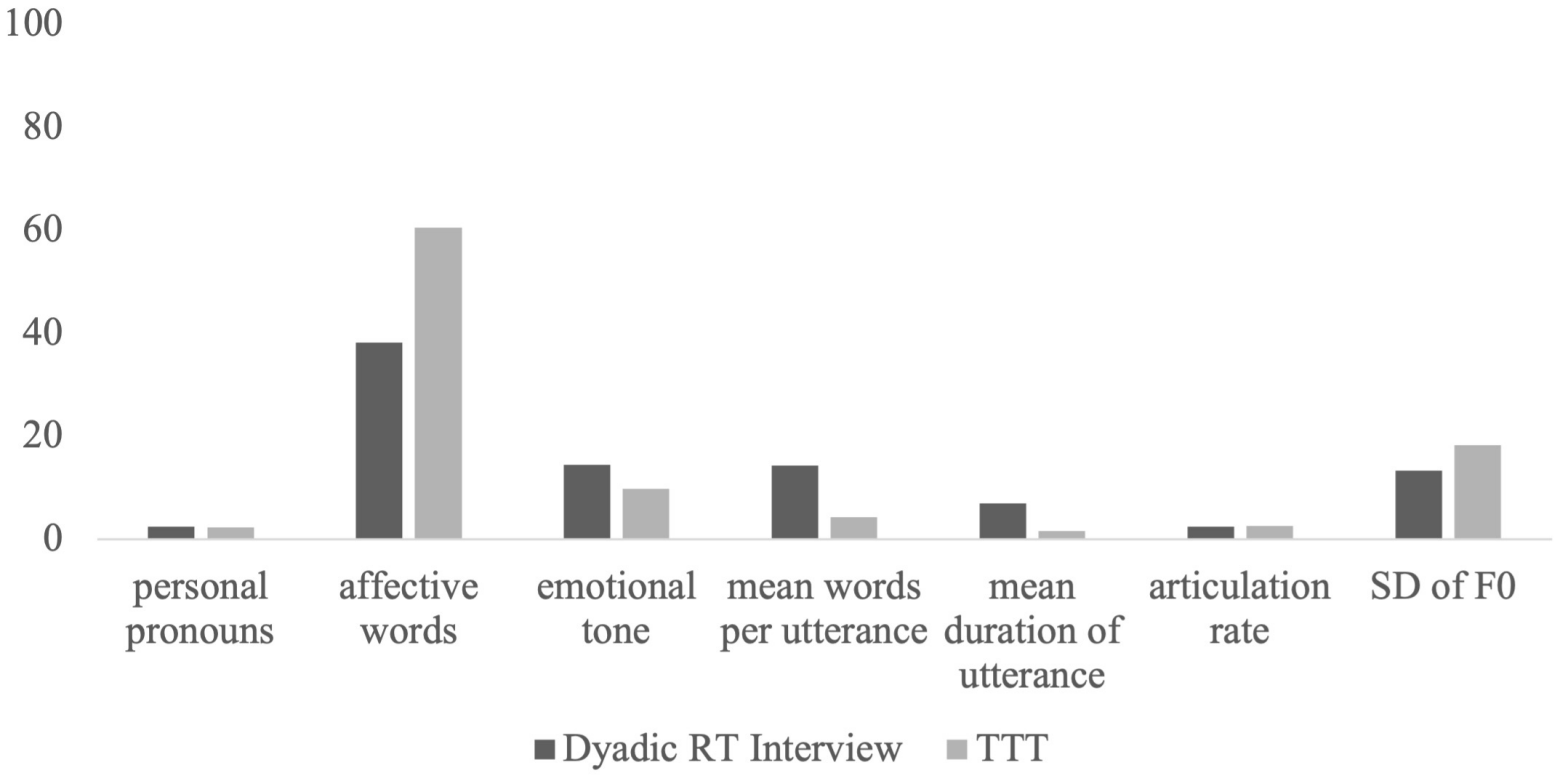
Mean of linguistic outcomes for Colette across two contexts: dyadic reminiscence therapy (RT) interview, and technology-driven group reminiscence therapy (TTT). The use of personal pronouns is measured as a percentage of all speech. Affective words incorporated both positive and negative emotive words and are measured as a percentagÍÍe of all speech. The emotional tone is measured from 0 (most negative) – 100 (most positive). The mean words per utterance is a numerical count value. The mean duration of utterance is measured in seconds. The articulation rate is the number of words spoken per second calculated from utterances. The standard deviation of fundamental frequency (SD of F0) is measured in Hertz.

Descriptively, as can be seen in Figure 7, the presence and intensity of AU04 for Colette were greater in the dyadic RT interview compared to the TTT setting. For AU06 there was greater presence of AU06 in the TTT context, however, there was greater intensity of AU06 in the dyadic RT interview. For all other AU’s, there was greater presence and intensity in the TTT context compared to the dyadic RT interview. Regarding the lexical use outcomes, as seen in Figure 8, Colette had greater personal pronoun use and a more positive emotional tone in the RT interview. However, there were more affective words used in the TTT context. Regarding the prosodic patterns, there was a greater mean words per utterance and mean duration of utterance in the RT interview and a high articulation rate and greater F0 variability in the TTT context.

## Discussion

The illustrations highlight the diversity of engagement and the analytical benefits of a multidimensional approach to characterize older adult engagement. If engagement was only measured with facial movement, a researcher might conclude greater engagement in the TTT context. However, the inclusion of prosodic patterns shows how there is greater verbal engagement in the dyadic RT interview. With the addition of the lexical use, we can get a greater insight into the affective experience of older adults. With the inclusion of a multidimensional approach and analysing facial movement as well as lexical use and prosodic patterns, a more nuanced set of measures of engagement is achieved.

While descriptive, the two illustrations of Angela and Colette demonstrate how different people may vary in engagement across the dimensions. When looking at facial movement as a measure of engagement, Angela and Colette show similar patterns of AU presence and intensity for AU04, AU12, AU15 and AU17. However, they differ in that Angela shows greater presence and reduced intensity of AU06 in the dyadic RT interview, and Colette shows a greater presence and reduced intensity of AU06 in the TTT context. When looking at prosodic features, both Angela and Colette show similar prosodic patterns. Both had a greater mean number of words per utterance and mean duration of utterance in the dyadic RT interview, and a faster articulation rate and greater F0 variability in the TTT context. When looking at lexical use as a measure of engagement, Angela and Colette show similar patterns of lexical use with increased personal pronoun use in the dyadic RT interview and increased affective word use in the TTT context. However, they differ in their emotional tone with Angela showing a more positive emotional tone in the TTT condition and Colette showing a more positive emotional tone in the dyadic RT interview.

These outcomes do not suggest that one measure of engagement is more important than another, or that the success of an intervention is reliant on a particular dimension revealing engagement. Rather, a multifactorial and dimensional approach provides a sensitive method to capture the impact of an intervention on an individual who may have varying capacities to engage. To understand the effectiveness of an intervention, this approach may be applied to a cohort of participants at baseline, and again during an intervention to determine the success of the intervention through the effect on engagement. That is, if a participant is displaying greater engagement across the dimensions during the intervention, as opposed to during the control situation, then the intervention may be deemed effective if engagement is a desired outcome. Similarly, it may also be a particularly useful tool when comparing two programs across participants and understanding their impact on engagement. This multidimensional approach gives researchers the ability to identify idiosyncratic outcomes of engagement. It allows for a greater understanding of how a psychosocial intervention affects different group members who may display engagement in different ways.

Future research plans are to apply this method to the TTT intervention across different residential care groups. With a greater sample size, we will apply mixed effects approaches to quantify the interindividual differences in the intraindividual engagement profiles. We will then be able to determine the impact of the TTT intervention on the engagement of older adults in residential care. In doing so, we will be able to characterize what facial, lexical and prosodic indicators of engagement are when cognitive profiles vary. By applying this multidimensional approach to different psychosocial interventions, we can begin to build an understanding of the repertoire or ways of how residents in aged care can and do engage across programs.

Since affect-driven engagement can be expressed through multiple behaviors, research can usefully consider adopting a multidimensional approach to measure engagement in older adults. The current paper outlines a method to more sensitively measure variations in individual behaviors and how interventions relate to engagement. This method is a useful tool to understand engagement of people with diverse profiles including dementia. The methods add to the ‘toolbox’ of researchers wishing to assess psychosocial interventions in naturalistic settings.

## Data availability statement

The datasets presented in this study can be found in online repositories. The names of the repository/repositories and accession number(s) can be found at: https://doi.org/10.26183/rb4c-ss12.

## Ethics statement

The studies involving human participants were reviewed and approved by Western Sydney University Human Ethics Committee. The patients/participants provided their written informed consent to participate in this study.

## Author contributions

MR contributed to conceptualization of framework and overarching research goals and aim, study design and data collection, application of analysis to PhD data, manuscript preparation and revision. WL contributed to the analysis method of the study and manuscript revision. CS contributed to conceptualization of framework and overarching research goals and aim, study design, data collection and manuscript revision. CH contributed to manuscript revision. CJ conceptualization of framework and overarching research goals and aim, study design and manuscript revision. All authors contributed to the article and approved the submitted version.

## Funding

The project was supported by funding to Western Sydney University as part of the Australian Research Council Centre of Excellence for the Dynamics of Language (ARC CoEDL, CE140100041), and a Western Sydney University PhD scholarship awarded to the first author. We acknowledge: BaptistCare Day Respite for collaborating with us on the Time Travelling with Technology Project; Andrew Leahy for technical assistance; and Deborah Parker for conceptual and supervisory contributions.

## Conflict of interest

The authors declare that the research was conducted in the absence of any commercial or financial relationships that could be construed as a potential conflict of interest.

## Publisher’s note

All claims expressed in this article are solely those of the authors and do not necessarily represent those of their affiliated organizations, or those of the publisher, the editors and the reviewers. Any product that may be evaluated in this article, or claim that may be made by its manufacturer, is not guaranteed or endorsed by the publisher.
