# Supplementary material for: Measuring engagement among older adults using a multidimensional approach to communication
Source: Front Psychol. 2022 Nov 21;13:981008. doi: 10.3389/fpsyg.2022.981008 (PMC9723878; doi:10.3389/fpsyg.2022.981008)
Supplement: Supplementary file 1 [file Data_Sheet_1.docx]

Supplementary Material

# Supplementary Figures


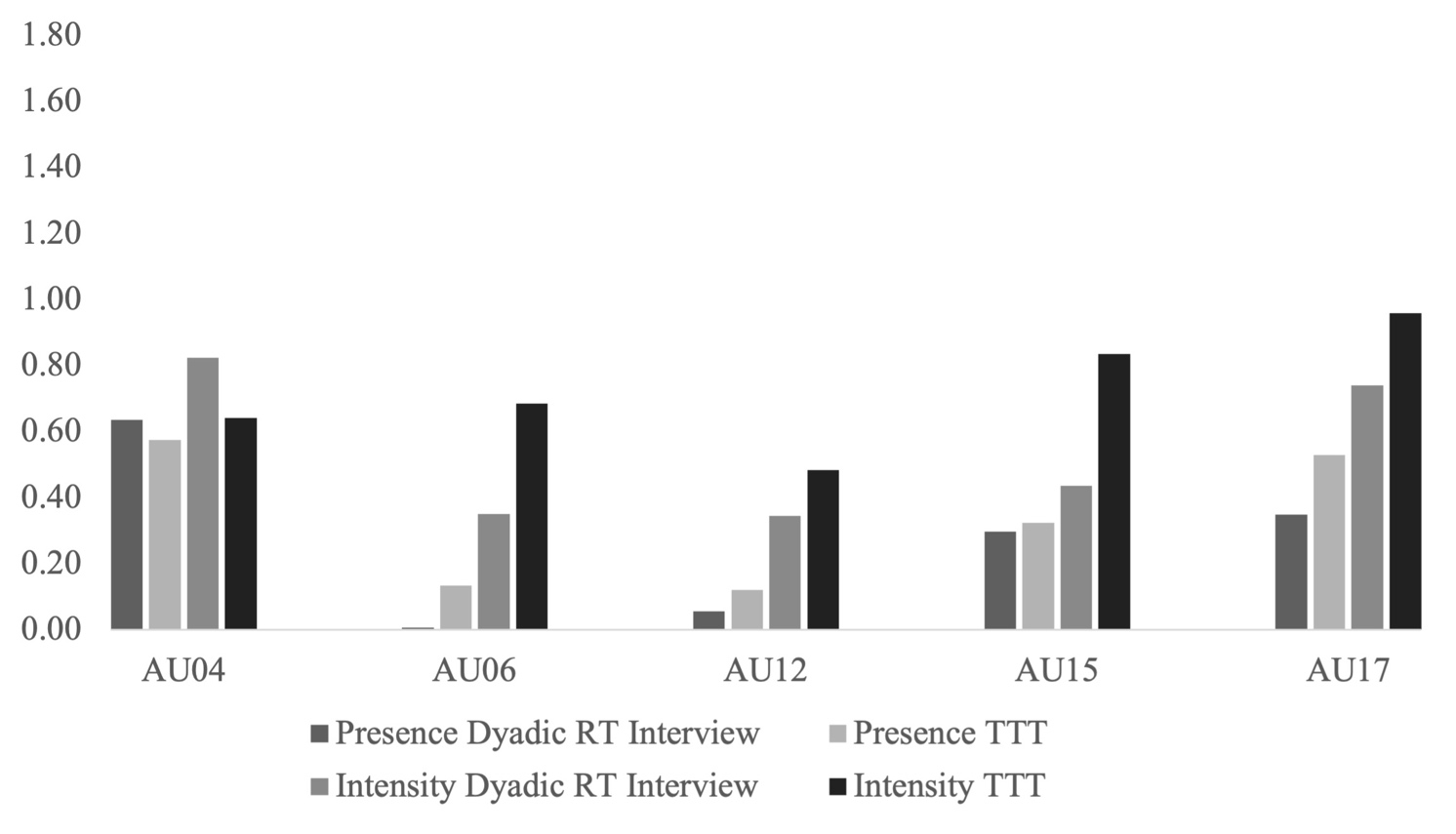


**Supplementary Figure 1.** Descriptive statistics of the behavior measures for Amy. Mean of action unit (AU) presence and intensity for Amy across two contexts: dyadic reminiscence therapy (RT) interview, and technology-driven group reminiscence therapy (TTT). The orange and green columns represent the RT context and the yellow and brown columns represent the TTT context. The presence of the AUs represents the percentage of time the AU was present from 0 (not present) to 1 (always present). The intensity of the AUs represents the intensity of the AU movement on a continuous scale from 0 (min) to 5 (max).


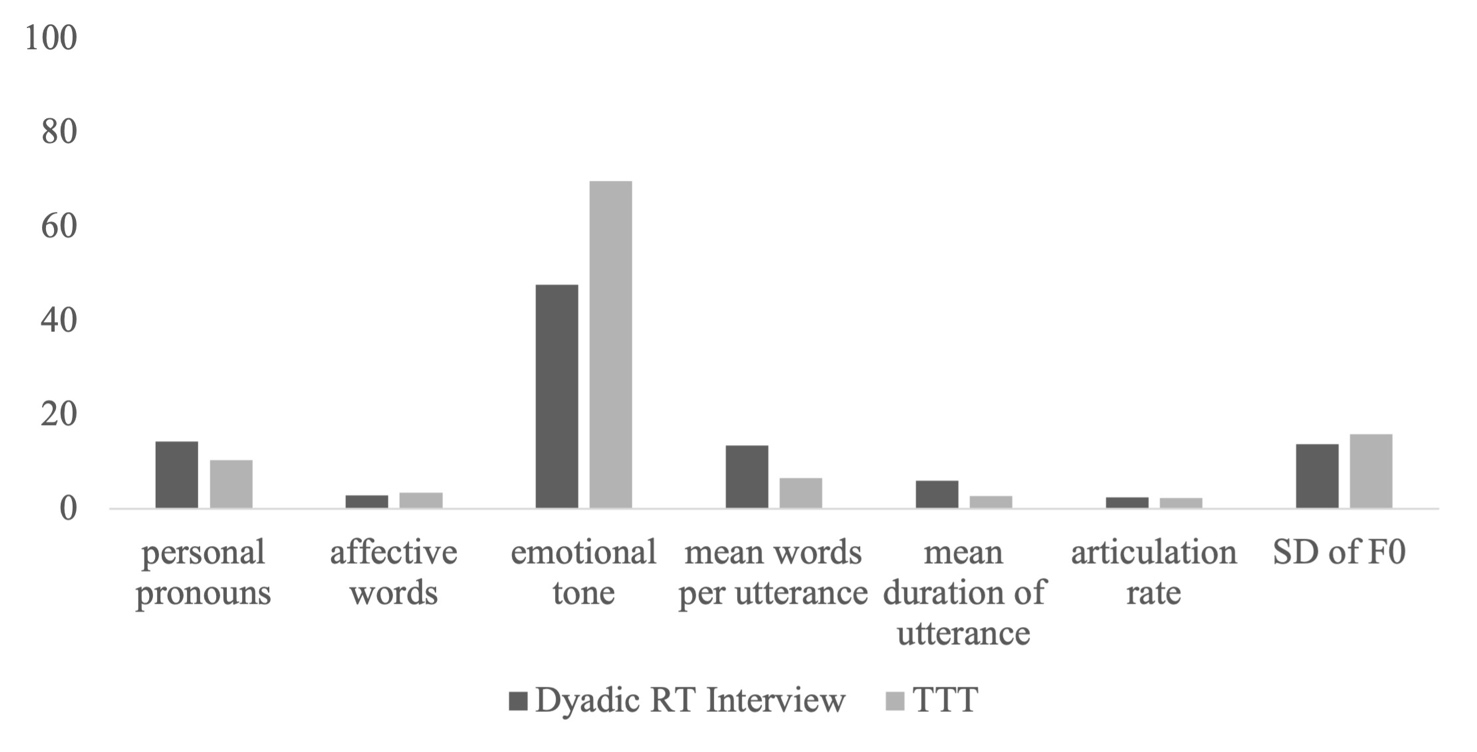


**Supplementary Figure 2.** Mean of linguistic outcomes for Amy across two contexts: dyadic reminiscence therapy (RT) interview, and technology-driven group reminiscence therapy (TTT). The use of personal pronouns is measured as a percentage of all speech. Affective words incorporated both positive and negative emotive words and are measured as a percentage of all speech. The emotional tone is measured from 0 (most negative) – 100 (most positive). The mean words per utterance is a numerical count value. The mean duration of utterance is measured in seconds. The articulation rate is the number of words spoken per second calculated from utterances. The standard deviation of fundamental frequency (SD of F0) is measured in Hertz.


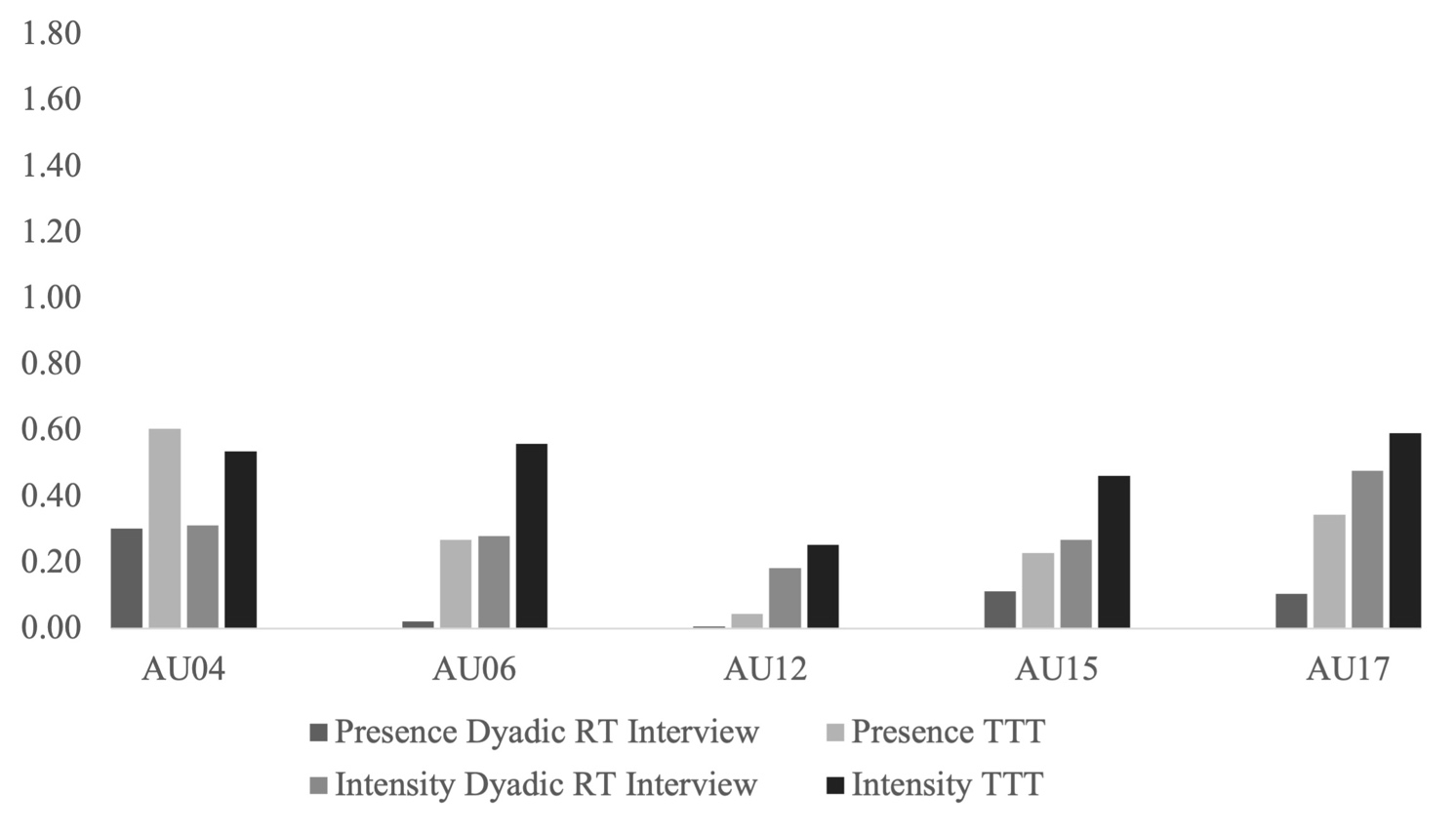


**Supplementary Figure 3.** Descriptive statistics of the behavior measures for Barbara. Mean of action unit (AU) presence and intensity for Barbara across two contexts: dyadic reminiscence therapy (RT) interview, and technology-driven group reminiscence therapy (TTT). The orange and green columns represent the RT context and the yellow and brown columns represent the TTT context. The presence of the AUs represents the percentage of time the AU was present from 0 (not present) to 1 (always present). The intensity of the AUs represents the intensity of the AU movement on a continuous scale from 0 (min) to 5 (max).


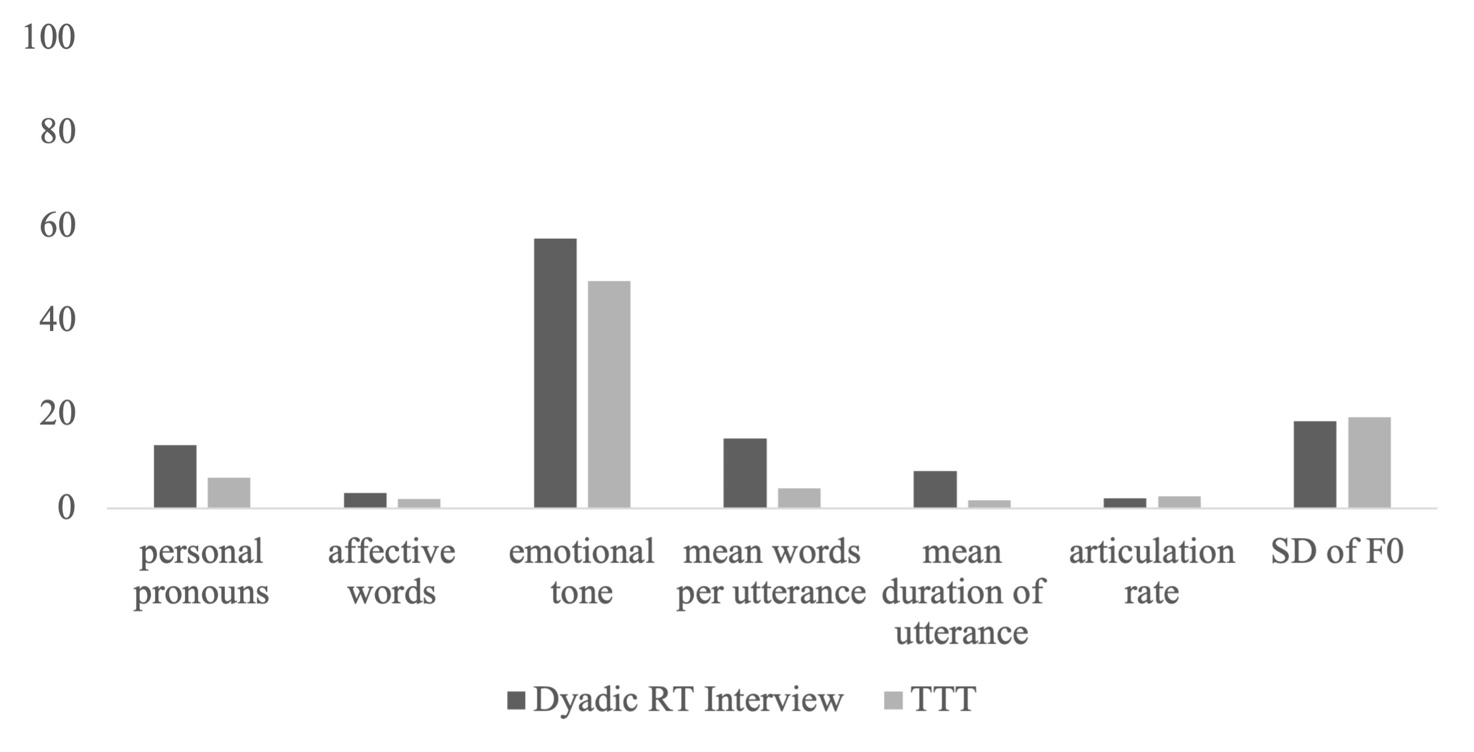


**Supplementary Figure 4.** Mean of linguistic outcomes for Barbara across two contexts: dyadic reminiscence therapy (RT) interview, and technology-driven group reminiscence therapy (TTT). The use of personal pronouns is measured as a percentage of all speech. Affective words incorporated both positive and negative emotive words and are measured as a percentage of all speech. The emotional tone is measured from 0 (most negative) – 100 (most positive). The mean words per utterance is a numerical count value. The mean duration of utterance is measured in seconds. The articulation rate is the number of words spoken per second calculated from utterances. The standard deviation of fundamental frequency (SD of F0) is measured in Hertz.


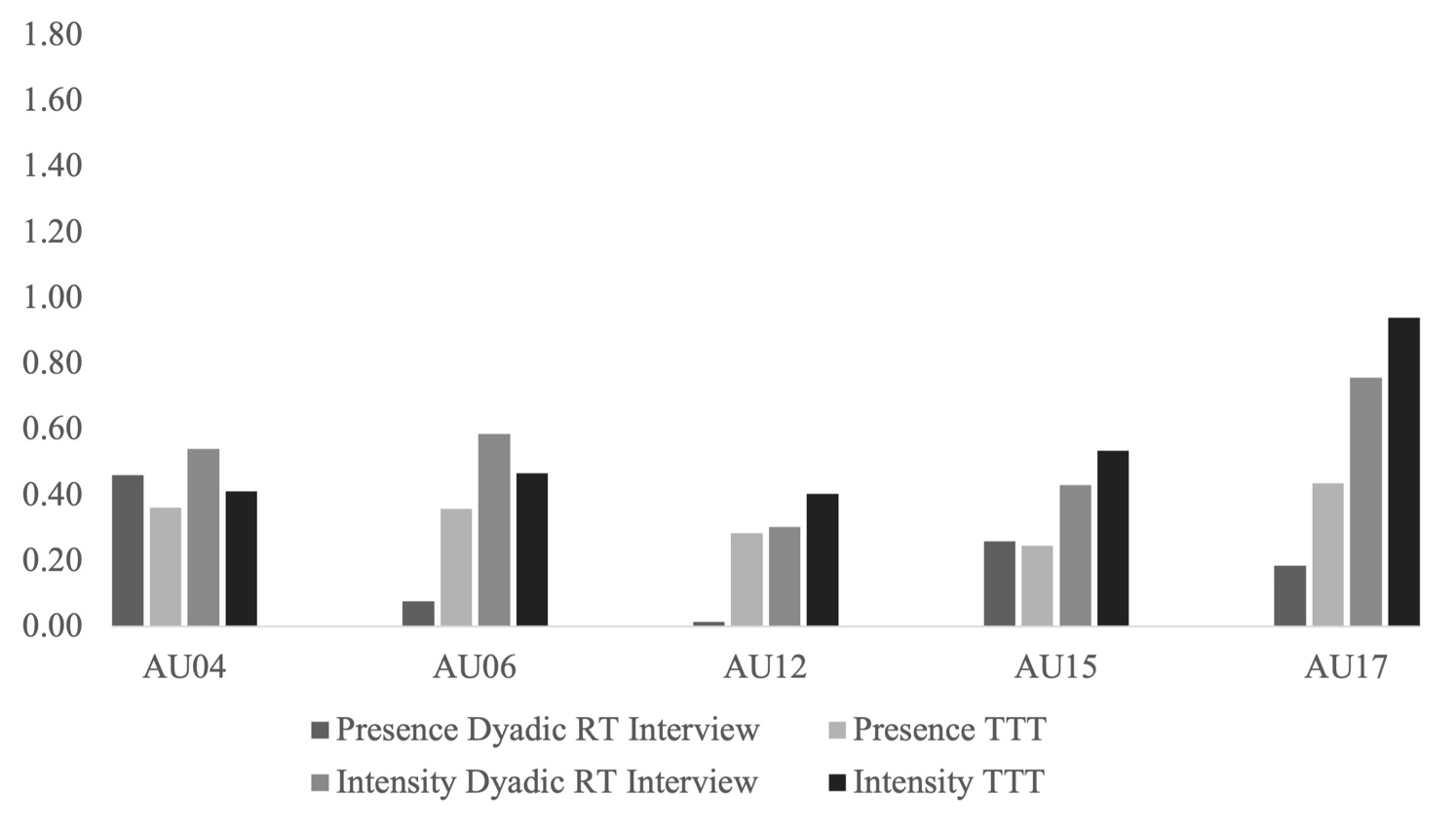


**Supplementary Figure 5.** Descriptive statistics of the behavior measures for Charlie. Mean of action unit (AU) presence and intensity for Charlie across two contexts: dyadic reminiscence therapy (RT) interview, and technology-driven group reminiscence therapy (TTT). The orange and green columns represent the RT context and the yellow and brown columns represent the TTT context. The presence of the AUs represents the percentage of time the AU was present from 0 (not present) to 1 (always present). The intensity of the AUs represents the intensity of the AU movement on a continuous scale from 0 (min) to 5 (max).


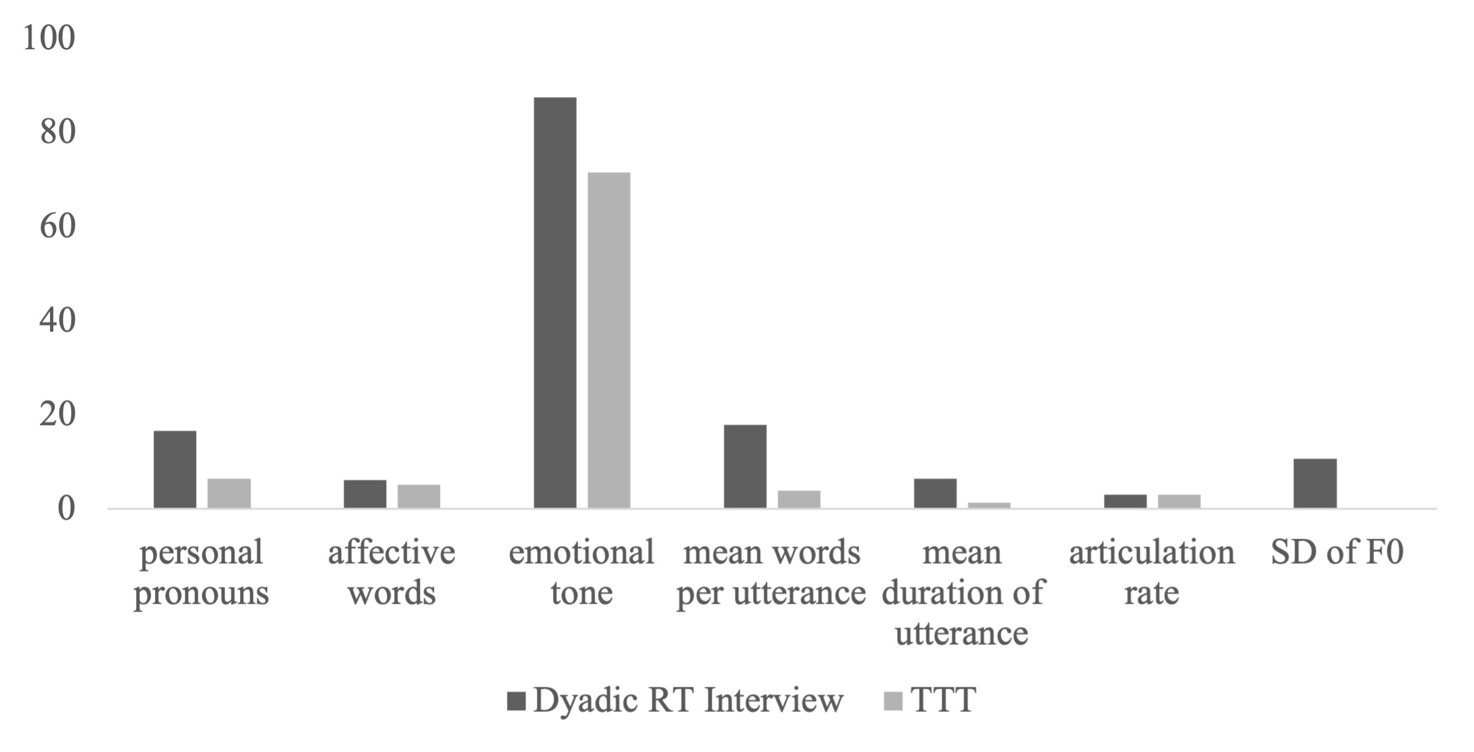


**Supplementary Figure 6.** Mean of linguistic outcomes for Charlie across two contexts: dyadic reminiscence therapy (RT) interview, and technology-driven group reminiscence therapy (TTT). The use of personal pronouns is measured as a percentage of all speech. Affective words incorporated both positive and negative emotive words and are measured as a percentage of all speech. The emotional tone is measured from 0 (most negative) – 100 (most positive). The mean words per utterance is a numerical count value. The mean duration of utterance is measured in seconds. The articulation rate is the number of words spoken per second calculated from utterances. The standard deviation of fundamental frequency (SD of F0) is measured in Hertz.


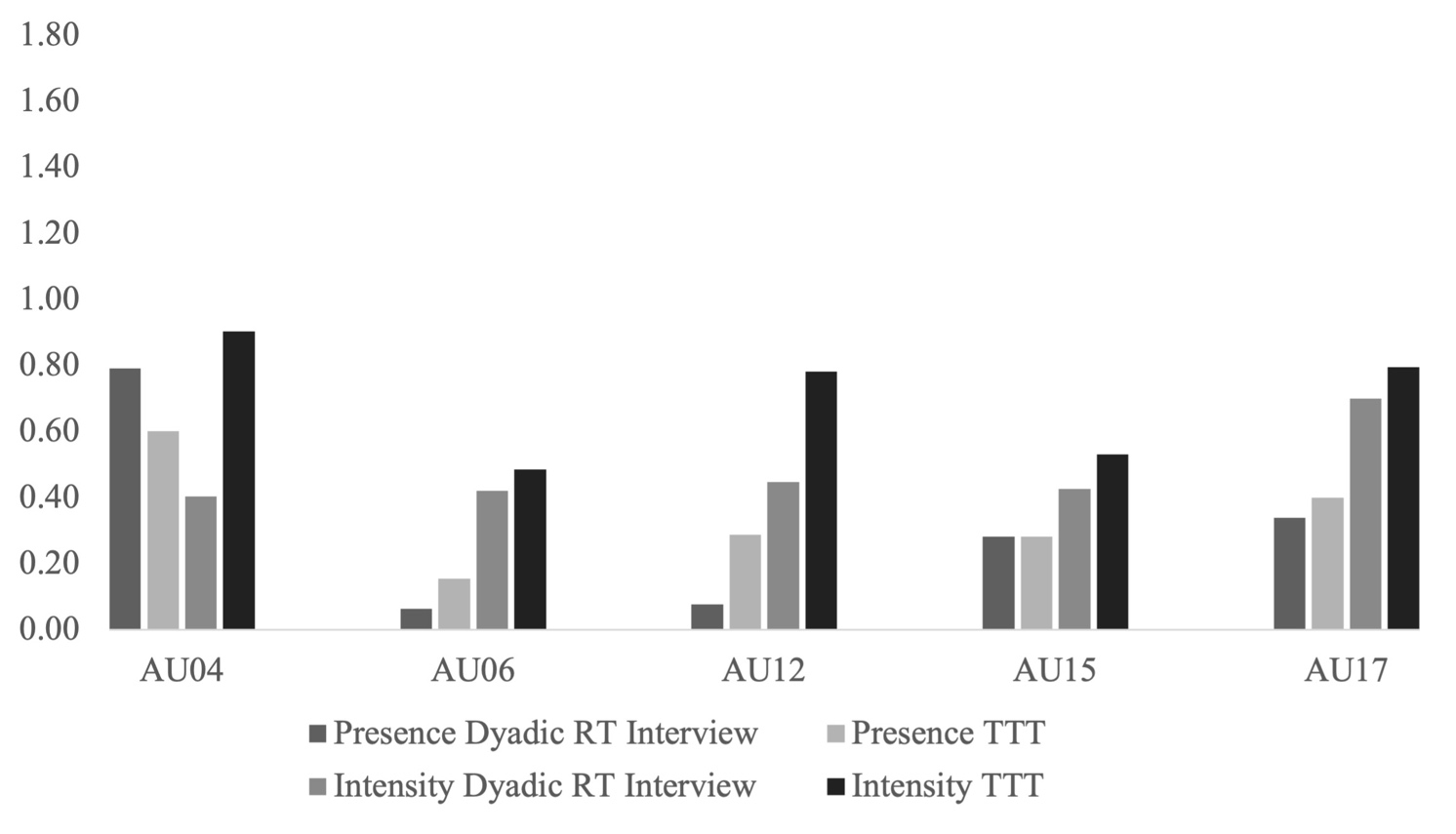


**Supplementary Figure 7.** Descriptive statistics of the behavior measures for Diana. Mean of action unit (AU) presence and intensity for Diana across two contexts: dyadic reminiscence therapy (RT) interview, and technology-driven group reminiscence therapy (TTT). The orange and green columns represent the RT context and the yellow and brown columns represent the TTT context. The presence of the AUs represents the percentage of time the AU was present from 0 (not present) to 1 (always present). The intensity of the AUs represents the intensity of the AU movement on a continuous scale from 0 (min) to 5 (max).


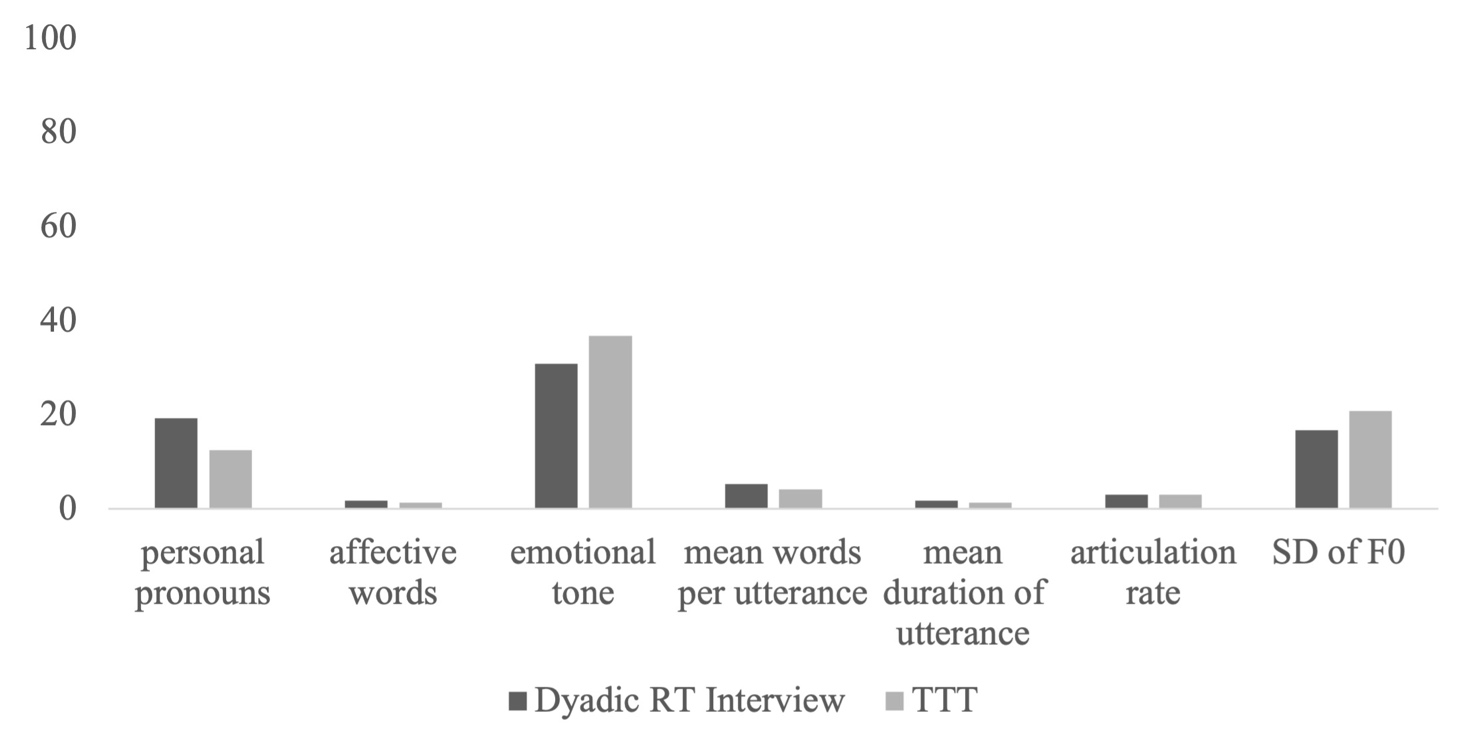


**Supplementary Figure 8.** Mean of linguistic outcomes for Diana across two contexts: dyadic reminiscence therapy (RT) interview, and technology-driven group reminiscence therapy (TTT). The use of personal pronouns is measured as a percentage of all speech. Affective words incorporated both positive and negative emotive words and are measured as a percentage of all speech. The emotional tone is measured from 0 (most negative) – 100 (most positive). The mean words per utterance is a numerical count value. The mean duration of utterance is measured in seconds. The articulation rate is the number of words spoken per second calculated from utterances. The standard deviation of fundamental frequency (SD of F0) is measured in Hertz.


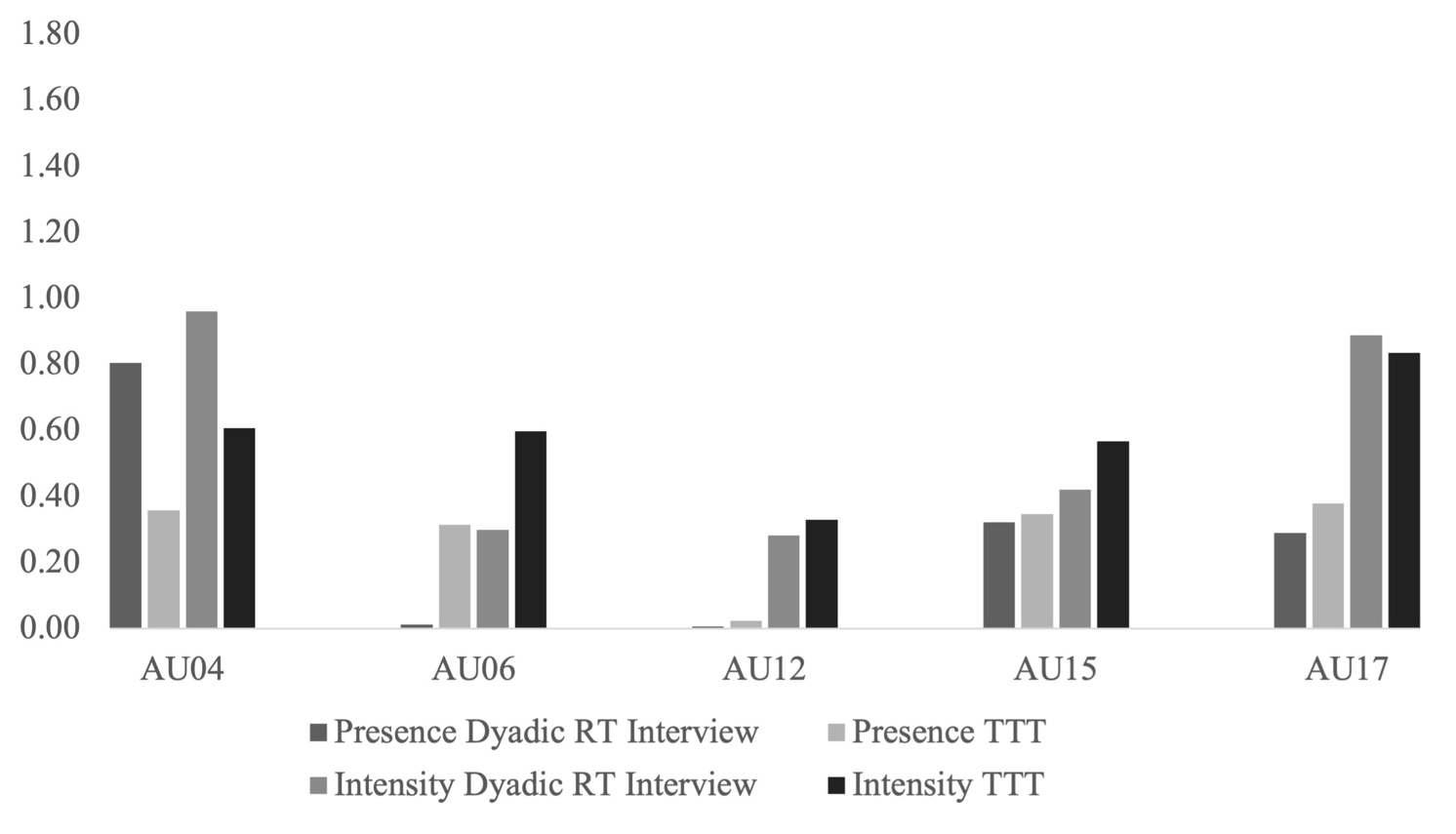


**Supplementary Figure 9.** Descriptive statistics of the behavior measures for Jana. Mean of action unit (AU) presence and intensity for Jana across two contexts: dyadic reminiscence therapy (RT) interview, and technology-driven group reminiscence therapy (TTT). The orange and green columns represent the RT context and the yellow and brown columns represent the TTT context. The presence of the AUs represents the percentage of time the AU was present from 0 (not present) to 1 (always present). The intensity of the AUs represents the intensity of the AU movement on a continuous scale from 0 (min) to 5 (max).


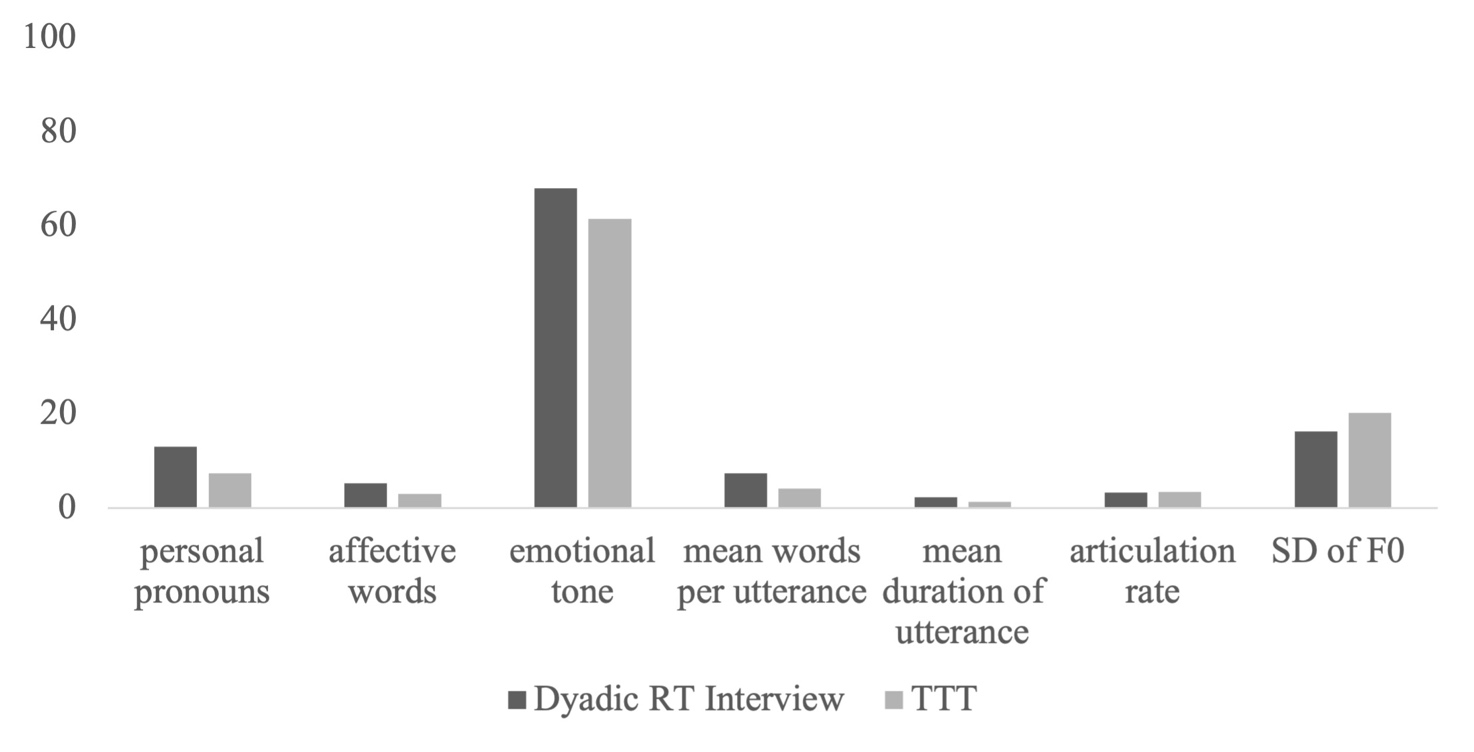


**Supplementary Figure 10.** Mean of linguistic outcomes for Jana across two contexts: dyadic reminiscence therapy (RT) interview, and technology-driven group reminiscence therapy (TTT). The use of personal pronouns is measured as a percentage of all speech. Affective words incorporated both positive and negative emotive words and are measured as a percentage of all speech. The emotional tone is measured from 0 (most negative) – 100 (most positive). The mean words per utterance is a numerical count value. The mean duration of utterance is measured in seconds. The articulation rate is the number of words spoken per second calculated from utterances. The standard deviation of fundamental frequency (SD of F0) is measured in Hertz.


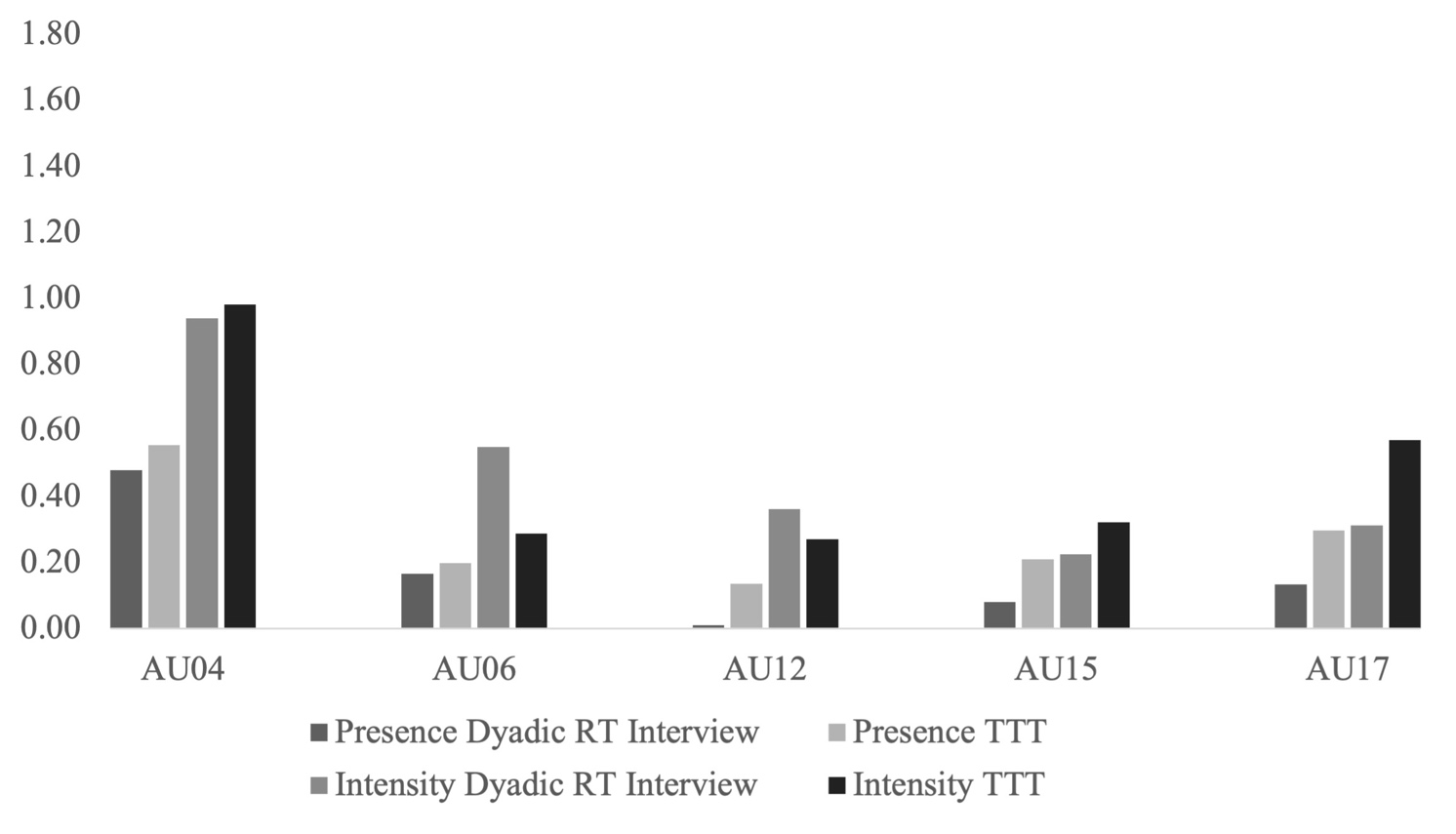


**Supplementary Figure 11.** Descriptive statistics of the behavior measures for Julia. Mean of action unit (AU) presence and intensity for Julia across two contexts: dyadic reminiscence therapy (RT) interview, and technology-driven group reminiscence therapy (TTT). The orange and green columns represent the RT context and the yellow and brown columns represent the TTT context. The presence of the AUs represents the percentage of time the AU was present from 0 (not present) to 1 (always present). The intensity of the AUs represents the intensity of the AU movement on a continuous scale from 0 (min) to 5 (max).


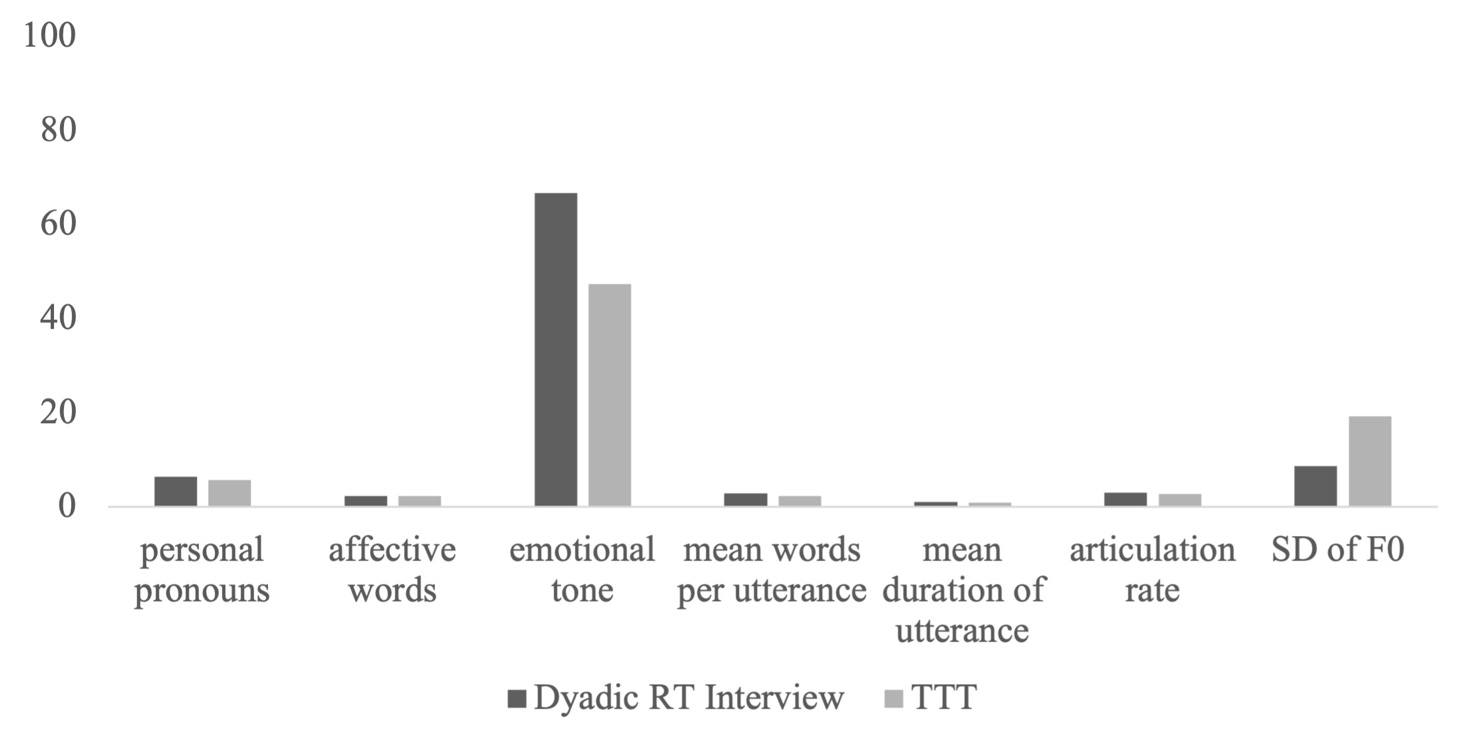


**Supplementary Figure 12.** Mean of linguistic outcomes for Julia across two contexts: dyadic reminiscence therapy (RT) interview, and technology-driven group reminiscence therapy (TTT). The use of personal pronouns is measured as a percentage of all speech. Affective words incorporated both positive and negative emotive words and are measured as a percentage of all speech. The emotional tone is measured from 0 (most negative) – 100 (most positive). The mean words per utterance is a numerical count value. The mean duration of utterance is measured in seconds. The articulation rate is the number of words spoken per second calculated from utterances. The standard deviation of fundamental frequency (SD of F0) is measured in Hertz.


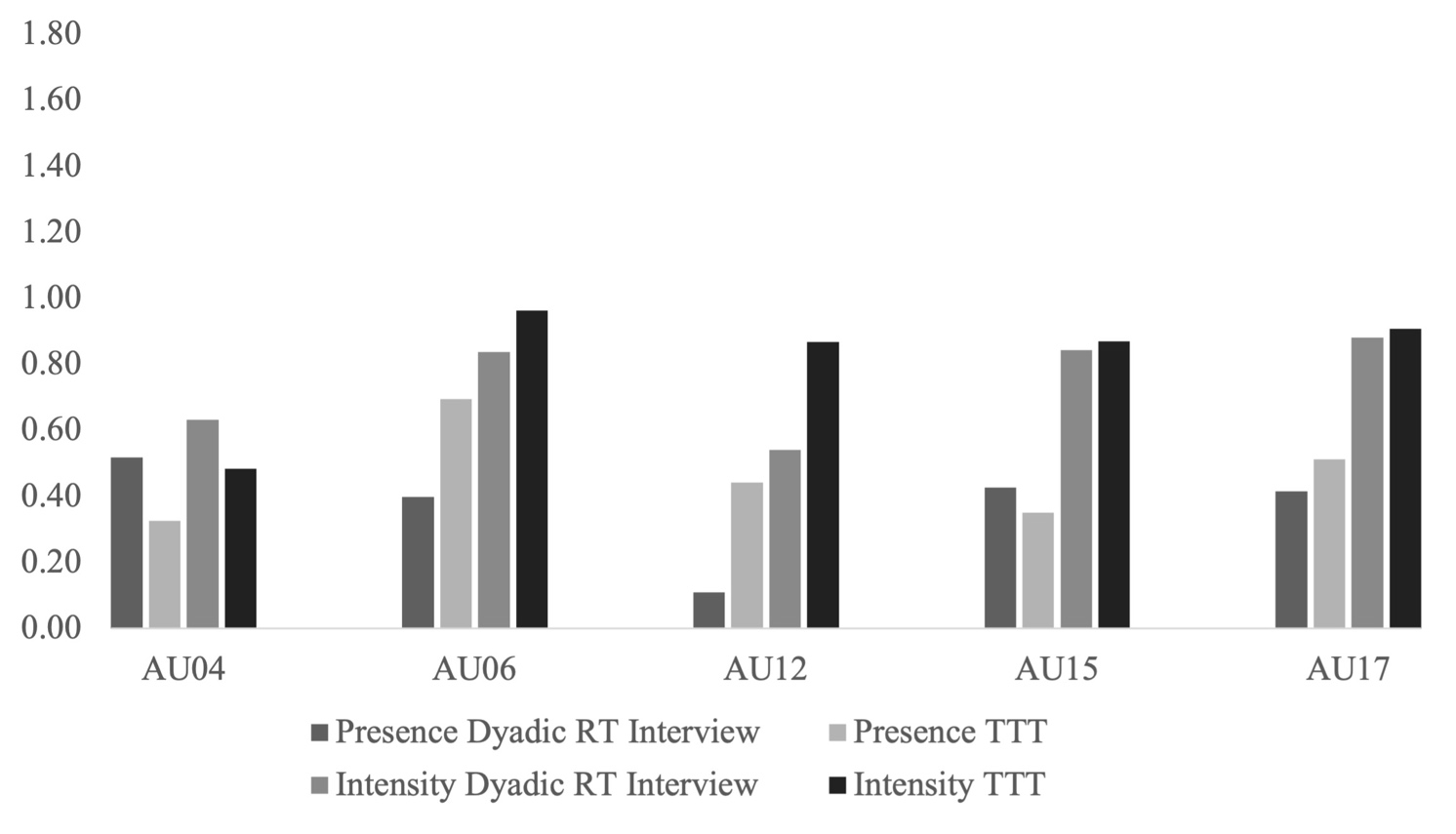


**Supplementary Figure 13.** Descriptive statistics of the behavior measures for Nora. Mean of action unit (AU) presence and intensity for Nora across two contexts: dyadic reminiscence therapy (RT) interview, and technology-driven group reminiscence therapy (TTT). The orange and green columns represent the RT context and the yellow and brown columns represent the TTT context. The presence of the AUs represents the percentage of time the AU was present from 0 (not present) to 1 (always present). The intensity of the AUs represents the intensity of the AU movement on a continuous scale from 0 (min) to 5 (max).


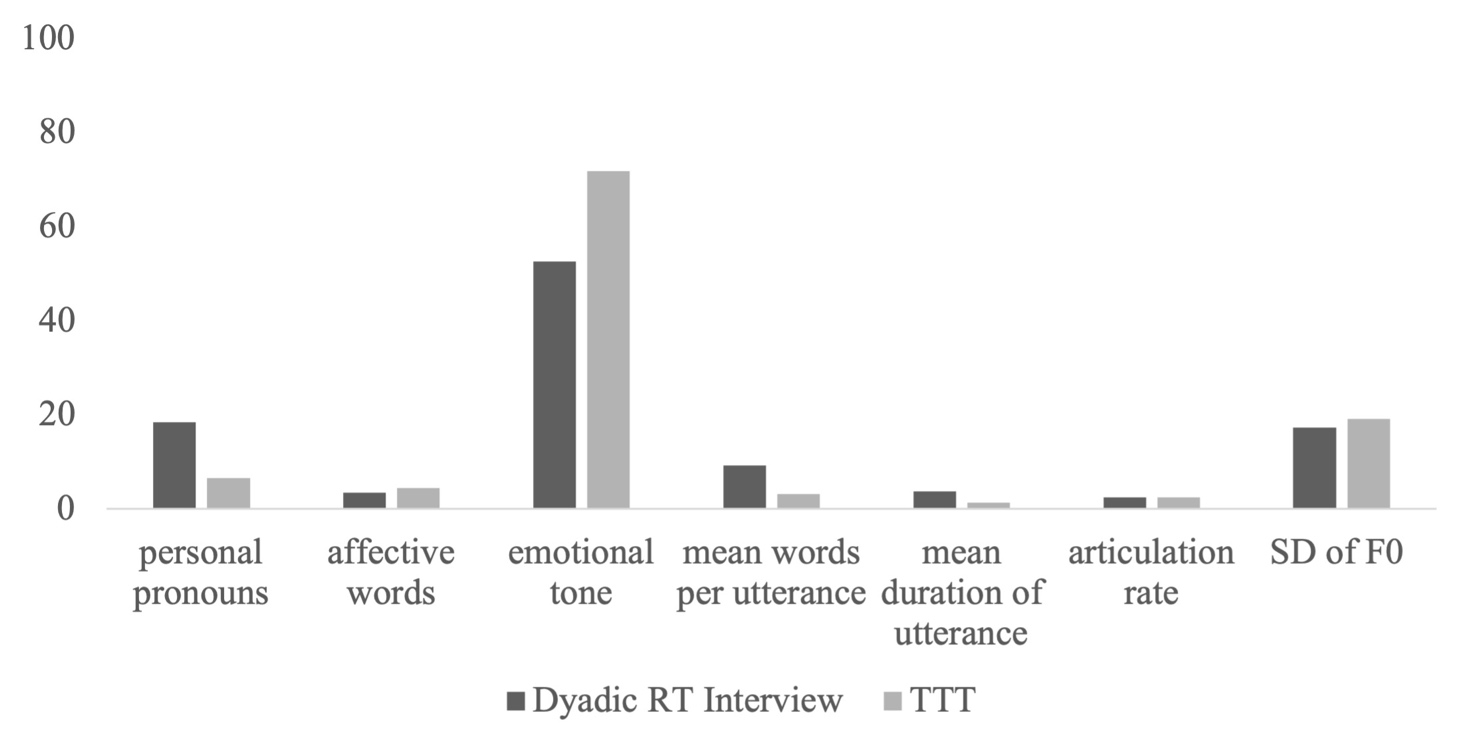


**Supplementary Figure 14.** Mean of linguistic outcomes for Nora across two contexts: dyadic reminiscence therapy (RT) interview, and technology-driven group reminiscence therapy (TTT). The use of personal pronouns is measured as a percentage of all speech. Affective words incorporated both positive and negative emotive words and are measured as a percentage of all speech. The emotional tone is measured from 0 (most negative) – 100 (most positive). The mean words per utterance is a numerical count value. The mean duration of utterance is measured in seconds. The articulation rate is the number of words spoken per second calculated from utterances. The standard deviation of fundamental frequency (SD of F0) is measured in Hertz.


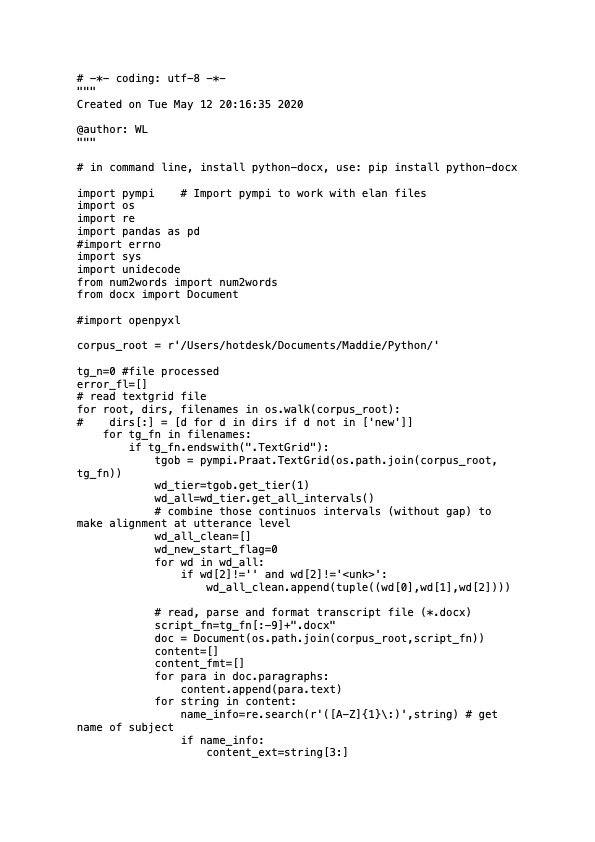


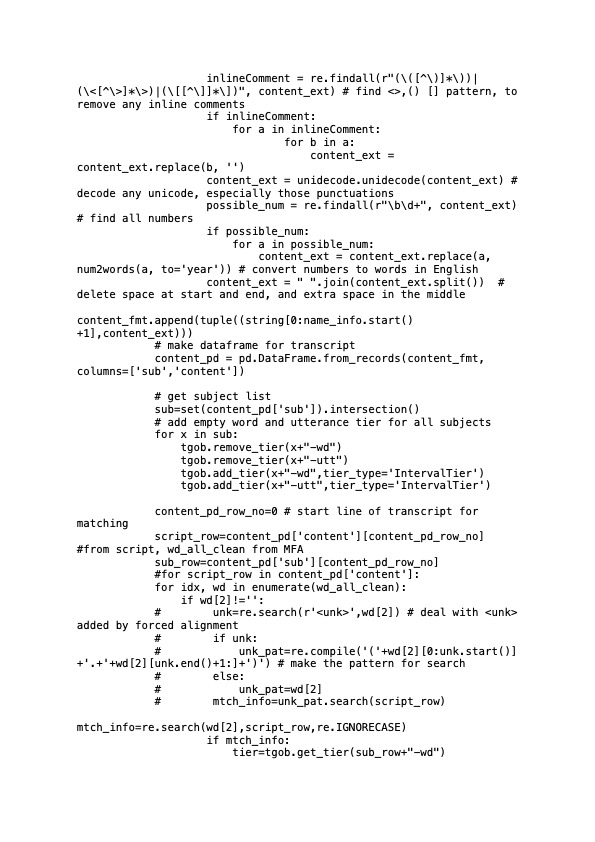


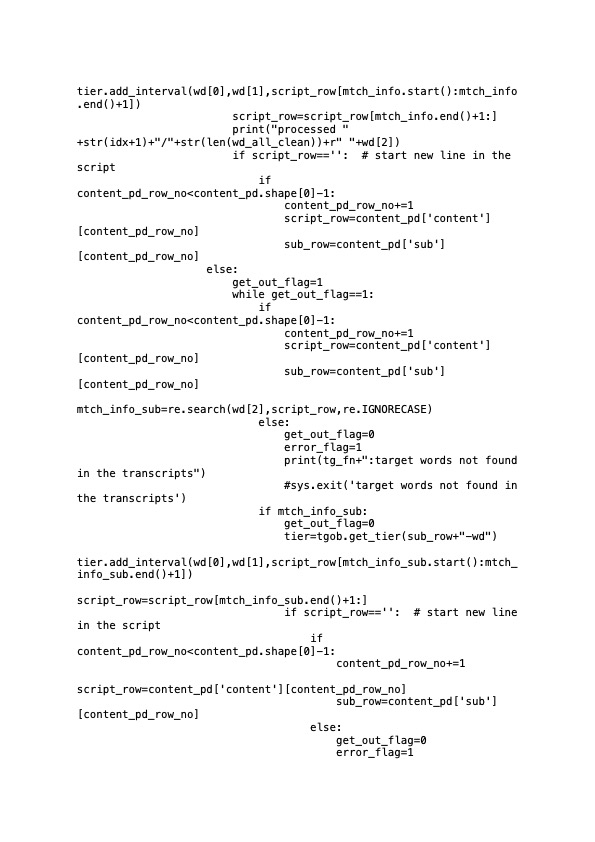


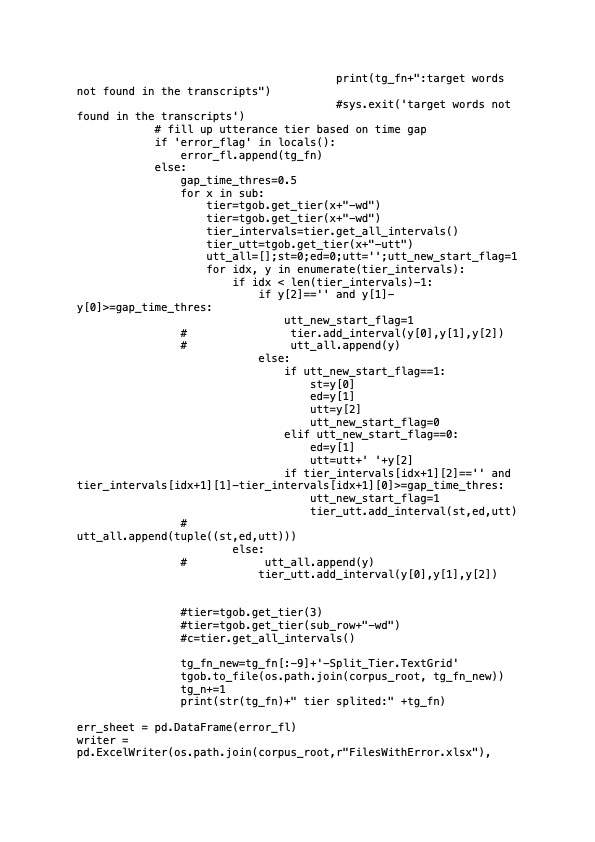

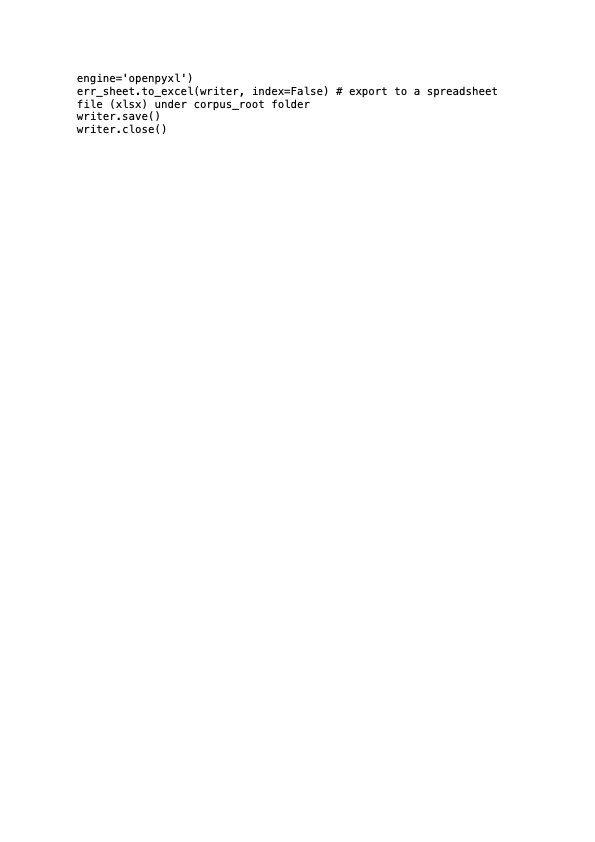


**Supplementary Figure 15.** Python script to reconstructed utterances using aligned words and assign utterances to individual speakers by comparing them with the corresponding transcript.


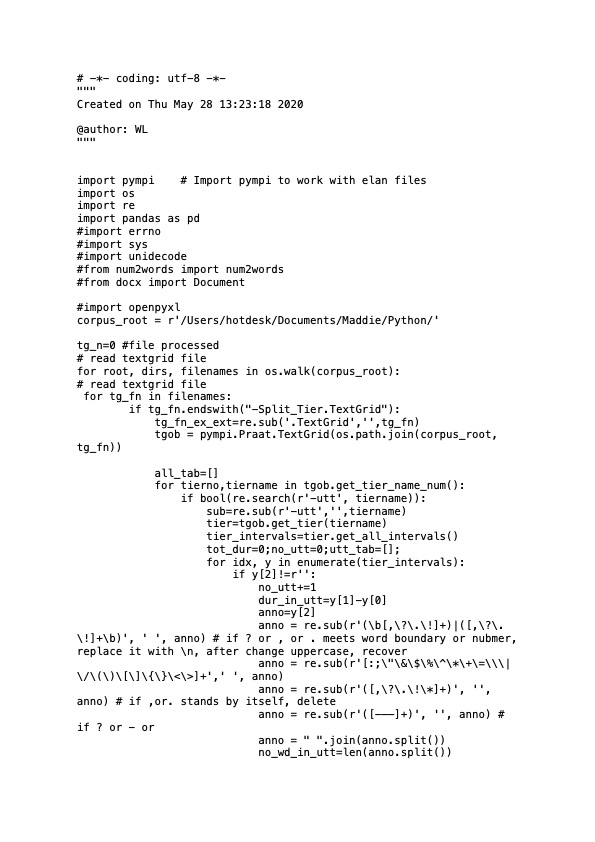


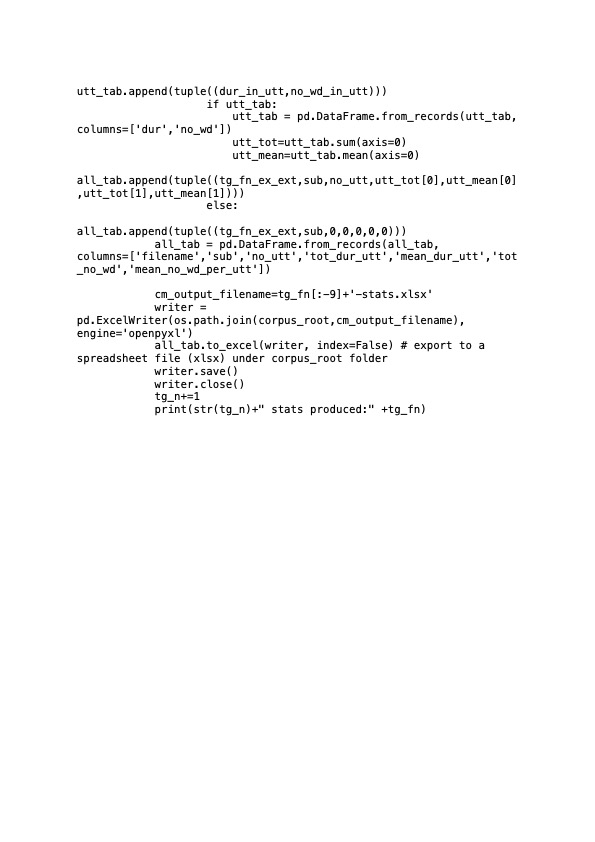


**Supplementary Figure 15.** Python script to extract measures of prosodic patterns from each speaker.Textgrid to a spreadsheet.
